# Age estimation from sleep studies using deep learning predicts life expectancy

**DOI:** 10.1038/s41746-022-00630-9

**Published:** 2022-07-22

**Authors:** Andreas Brink-Kjaer, Eileen B. Leary, Haoqi Sun, M. Brandon Westover, Katie L. Stone, Paul E. Peppard, Nancy E. Lane, Peggy M. Cawthon, Susan Redline, Poul Jennum, Helge B. D. Sorensen, Emmanuel Mignot

**Affiliations:** 1 Department of Health Technology, Technical University of Denmark, Kongens Lyngby, Denmark; 2 Danish Center for Sleep Medicine, Department of Clinical Neurophysiology, Rigshospitalet, Denmark; 3 Stanford Center for Sleep Sciences and Medicine, Stanford University, Palo Alto, CA USA; 4 Department of Neurology, Massachusetts General Hospital, Boston, MA USA; 5 Research Institute, California Pacific Medical Center, San Francisco, CA USA; 6 Department of Epidemiology and Biostatistics, University of California, San Francisco, CA USA; 7 Department of Population Health Sciences, University of Wisconsin-Madison, Madison, WI USA; 8 Department of Medicine, University of Davis School of Medicine, Sacramento, CA USA; 9 Department of Medicine, Harvard Medical School, Boston, MA USA; 10 Department of Medicine, Brigham and Women’s Hospital, Boston, MA USA

**Keywords:** Neural ageing, Prognostic markers, Risk factors, Predictive markers

## Abstract

Sleep disturbances increase with age and are predictors of mortality. Here, we present deep neural networks that estimate age and mortality risk through polysomnograms (PSGs). Aging was modeled using 2500 PSGs and tested in 10,699 PSGs from men and women in seven different cohorts aged between 20 and 90. Ages were estimated with a mean absolute error of 5.8 ± 1.6 years, while basic sleep scoring measures had an error of 14.9 ± 6.29 years. After controlling for demographics, sleep, and health covariates, each 10-year increment in age estimate error (AEE) was associated with increased all-cause mortality rate of 29% (95% confidence interval: 20–39%). An increase from −10 to +10 years in AEE translates to an estimated decreased life expectancy of 8.7 years (95% confidence interval: 6.1–11.4 years). Greater AEE was mostly reflected in increased sleep fragmentation, suggesting this is an important biomarker of future health independent of sleep apnea.

## Introduction

Sleep clinics throughout the world evaluate millions of patients every year. The gold standard diagnostic test for this evaluation is nocturnal polysomnography (PSG), a test comprised of multiple physiological signals, i.e., electroencephalogram (EEG), electrocardiogram (ECG), electrooculogram (EOG), chin and leg electromyogram (EMG), breathing effort and airflow, all of which are recorded overnight. The PSG provides recording of multiple physiological measures during sleep, at a time when the individual is mostly immobile and uncontaminated by sensory inputs. It thus contains a wealth of information on the normal physiology of a given individual (notably brain physiology).

Sadly, the millions of PSGs collected every year are primarily used clinically to visually extract simple metrics such as sleep latency, proportion of time in various sleep stages, rates of sleep apnea events (apnea-hypopnea index, AHI), periodic leg movement (PLM), and arousals (arousal index, ArI). Scoring is done manually by trained technicians and supervised by medical doctors, according to American Academy of Sleep Medicine (AASM) guidelines^1^. This scoring is time-consuming and prone to inter- and intra-rater variability^2^. Of particular clinical importance are measures of sleep disordered breathing events such as the AHI or associated hypoxic burden, which has been associated with daytime sleepiness^3^, cognitive impairment, and increased risk of cardiovascular disease such as development of high blood pressure and stroke in multiple studies independent of age, sex and obesity^4–9^. Sleep apnea has also been shown to be associated with increased mortality risk independent of obesity, age, and sex^10^.

Although sleep apnea measures are currently the main rationale for conducting clinical sleep studies, there is evidence that other aspects of objective sleep influence mortality and health outcomes. All-cause mortality has been associated with an increase in arousal burden^11^ (a measure of sleep fragmentation), decreased sleep efficiency (SE)^12^ and decreased rapid eye movement (REM) sleep amounts^13^. Similarly, decreased slow-wave sleep and low SE have been associated with hypertension incidence and a variety of cardiovascular outcomes among participants in the Sleep Heart Health Study (SHHS)^14,15^. Finally, specific abnormalities such as REM sleep behavior disorder (RBD) and loss of sleep-stage specific autonomic regulation during sleep are well established early precursors of synucleinopathies^16–18^.

Recently, promising deep learning methods have been developed that efficiently and objectively assist PSG analyses^19–21^. These algorithms provide added information such as higher resolution sleep stages and probabilistic measures, in contrast to manual scoring that only offers categorical classification. However, these new methods have mostly been confined to replicating a scoring practice that is limited by arbitrary definitions^1^ that may not capture all relevant information available in the data. Further, they merely imitate human scoring without attempting to capture all the rich incipient information contained in a full night PSG study discussed above. Deep learning methods that utilize all relevant information in PSGs may provide additional useful clinical insights such as important health outcomes.

Age is one of the strongest predictors of morbidity and mortality. Sleep architecture and subjective sleep complaints are also affected by aging^22,23^. As people age, sleep becomes shorter^23^, more fragmented^24^, exhibits fewer sleep spindles^25^, includes less slow wave sleep, and, to a lesser extent, less REM sleep^26^. Moreover, several of these changes have been linked to increased mortality, even after controlling for the effects of age^11–13^.

A recent study modeled the age of subjects based on automatic sleep-stage features from EEG recordings^27^. Furthermore, this model’s age estimate (AE) error (AEE), the model residual that represented a brain aging index, was associated with increased risk of mortality^28^, dementia^29^, and human immunodeficiency virus (HIV)^30^. However, as the authors pointed out, this approach was still limited by the use of hand-crafted features, and used only the EEG signal, whereas other physiological signals also carry important information about health and life expectancy. Nonetheless, since age is readily available in all subjects, (unlike mortality or other outcomes) predicting age may be a reasonable first proxy to predicting poor outcomes in a variety of disease area.

In this study, we built on this previous study aiming by (1) modeling age, as a proxy for mortality risk, directly using deep learning models; (2) interpreting the features learned by the models; and (3) investigating associations between the AEE of the models and both all-cause and cardiovascular mortality.

## Results

### Performance of age estimation models

In this study, we used a combined sample of 13,332 PSGs from seven cohorts: the Stanford Technology Analytics and Genomics of Sleep (STAGES)^31,32^, the Stanford Sleep Cohort (SSC)^33,34^, the Wisconsin Sleep Cohort (WSC)^4,34^, the SHHS^35,36^, the Osteoporotic Fractures in Men (MrOS) Sleep Study^36–38^, the Cleveland Family Study (CFS)^36,39^, and the Home Positive Airway Pressure (HomePAP) Study^36,40^.

A set of AE models, comprised of deep neural networks, were trained on 2500 PSGs from subjects with a close to uniform age distribution between 6 and 90 years. These AE models each used a set of input PSG signals: (a, Central EEG) C3-M2, C4-M1; (b, EEG + EOG + EMG) C3-M2, C4-M1, L-EOG, R-EOG, chin EMG; (c, ECG) ECG; (d, respiratory) airflow, nasal pressure, thoracic and abdominal belts, blood oxygen saturation. Finally, an ensemble model (e, Ensemble–Avg.) was developed based on the average AE of models (a), (b), (c), and (d). A validation set of 200 PSGs were used to optimize hyperparameters of the AE models, of which the final hyperparameter tunings are shown in Supplementary Table 1.

Performance of various AE models (based on EEG alone or various components of the PSG, see Table 1) was evaluated as mean absolute error (MAE) stratified by 5-year age intervals, as shown in Supplementary Table 2 for the first test set, and in Supplementary Table 3 for the HomePAP study (a second test dataset with an age range from 20 to 80 years). The stratification weighs each age interval equally despite a non-uniform age distribution. Table 1 shows MAE for each data subset averaged across all 5-year age groups ranging from 20 to 90 years. The best performing model on the test set was the (e, Ensemble–Avg.) model, which averages model (a–d), while the (a, Central EEG) model generalized best to the HomePAP dataset. As a comparison, we also report performance of using basic sleep study metrics for age estimation, which includes ArI, AHI, total sleep time (TST), wake after sleep onset (WASO), and percentage of NREM stages (N1, N2, N3), and REM sleep.

**Table 1:** Mean absolute error of age estimation models.

	MAE			
Model	Train set *n* = 2500	Val set *n* = 200	Test set *n* = 10,509	HomePAP* *n* = 190
Basic sleep measures	14.9 ± 6.08	14.9 ± 6.53	14.6 ± 5.91	12.5 ± 4.06
(a) Central EEG	5.43 ± 1.25	6.52 ± 2.48	6.77 ± 2.2	7.65 ± 2.7
(b) EEG+EOG+EMG	5.35 ± 0.96	5.88 ± 2.09	6.81 ± 1.84	8.62 ± 2.92
(c) ECG	9.11 ± 1.89	11 ± 4.05	10.4 ± 2.23	13.9 ± 6.74
(d) Respiratory	8.87 ± 2.2	9.31 ± 2.39	8.09 ± 1.89	13.7 ± 6.05
(e) Ensemble–Avg.	5.4 ± 1.01	6.11 ± 1.84	5.8 ± 1.16	8.16 ± 3.75

The MAE is reported as mean ± standard deviation and was averaged across age intervals ([20, 25], [25, 30], …, [85–89]), which are reported for the test and HomePAP set in Supplementary Tables 2 and 3. *The training and validation set includes no PSGs from the HomePAP study, thus it represents expected performance in a new unseen cohort with a different technical setup. Basic sleep measures denote a linear regression model with the following predictive variables: arousal index, apnea-hypopnea index, total sleep time, wake after sleep onset, and percentage of N1, N2, N3, and REM sleep. MAE: mean absolute error.

**Fig. 1:**
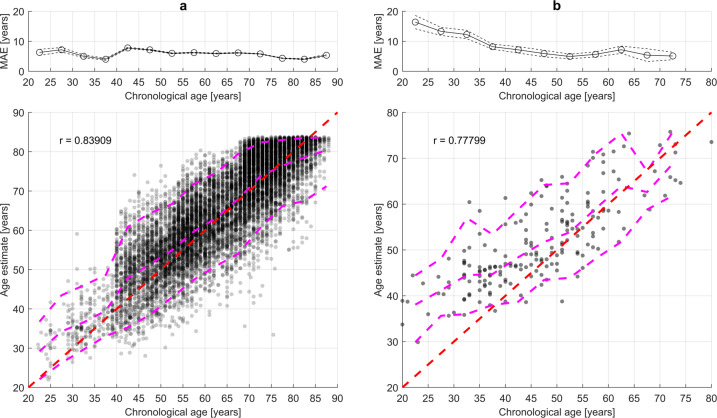
Scatterplot of age estimate and chronological age in the test sets for model (e, Ensemble–Avg.). **a** The test set (*n* = 9899). The dotted line indicates the standard error of the mean (SEM) calculated as $\sigma /\sqrt n$. **b** The HomePAP test set (*n* = 190). The red line indicates the optimal age estimate; the magenta lines indicate 5th, 50th, and 95th percentiles of age estimate in 5-year intervals. r is Pearson’s correlation coefficient between age estimate and chronological age. MAE: mean absolute error.

A scatterplot of AE for model (e, Ensemble–Avg.) and chronological age for the test set and HomePAP data is shown in Fig. 1.

Night-to-night variability was investigated in the STAGES dataset (*n* = 42). MAE was 5.93 years and 7.31 years during night 1 and 2, respectively. The difference between night 2 and 1 was −1.17 ± 5.71 (mean ± standard deviation), which was not significantly different from 0 (*p* = 0.19). The absolute difference between nights were 4.42 ± 3.74 years (*p* = 2∙10^−9^).

The reliability of the AEs in longitudinal data was investigated in the WSC (*n* = 505) with a time of 4.08 ± 1.02 years between visits. The MAE was 4.34 ± 3.07 years for the first visit and 4.51 ± 3.32 years for the second visit. The AE increased by 3.37 ± 4.05 between visits. Hence, the average increase was 0.7 years higher than for the chronological age (*p* = 0.00016).

### Interpretation of deep learning framework and of age estimation errors

The age difference obtained between the various AE models and chronological age, i.e., AEE, can be considered a measure of how much “younger” or “older” sleep in a PSG appears. As a sanity check, we first examined associations between AEE of the models with basic sleep measures, which are shown in Supplementary Table 4. In general, higher AEE was associated with worse sleep based on metrics related to sleep fragmentation [ArI, SE, WASO, TST, and N1%]. The respiratory-based AEE shows a very strong association with the AHI (*b* = 1.5, *p* = 4.7∙10^−76^), suggesting that it indeed captures information about sleep disordered breathing, which is known to increase with age.

Associations between AEE and sex, body mass index (BMI), medication use (antidepressants and benzodiazepines), and morbidities [hypertension, history of heart attack, congestive heart failure (CHF), chronic obstructive pulmonary disease (COPD), type 2 diabetes (T2D), and stroke] are shown in Supplementary Table 5. Presence of T2D was associated with a higher AEE (*b* = 1.6, *p* = 9.0∙10^−7^) for the (a, Central EEG) model and (*b* = 1.2, *p* = 7.7∙10^−5^) for the (b, EEG + EOG + EMG) model. For the (c, ECG) model, all heart related comorbidities were associated with a higher AEE (hypertension: 2.2 years, *p* = 8.8∙10^−24^; CHF: 3.1 years, *p* = 5.1∙10^−7^; history of heart attack: 1.8 years, *p* = 3.3∙10^−6^). Moreover, hypertension was associated with higher AEE in all but the (d, respiratory) model. Sex and BMI was associated with higher AEE in the (d, respiratory) model (sex: *b* = 3.6, *p* = 3.5∙10^−96^; BMI: *b* = 1.2, *p* = 2.0∙10^−54^). As for the stroke, COPD, and use of benzodiazepines, no significant associations to AEE were found.

**Fig. 2:**
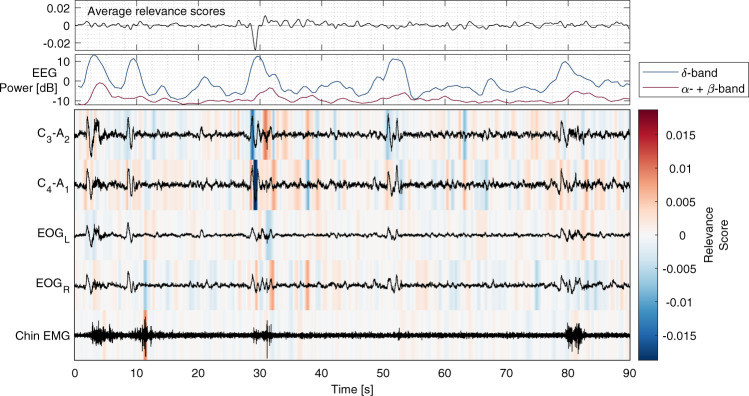
Example of model (b; EEG+EOG+EMG) interpretation through relevance attribution of samples. The top plot shows relevance scores averaged across channels (C3-A2, C3-A1, EOGL, EOGR, Chin EMG). The second plot shows EEG power in the δ-band (0–4 Hz) and the combined α- and β-bands (>8 Hz). Red and blue indicate positive and negative attribution to the age estimate, respectively. Relevance attribution was computed using gradient SHAP.

Gradient SHAP^41,42^ (SHapley Additive exPlanations) was used to attribute relevance scores of the AE to input PSG signal samples. For a given PSG, each signal sample was attributed with a relevance score that add up the AE for all samples in that PSG. Visual interpretation of relevance attribution, as shown in Fig. 2, shows that model (b, EEG + EOG + EMG) AE is increased in the presence of arousals and decreased in the presence of slow-wave oscillations. Furthermore, as shown in Supplementary Fig. 1, model (d, respiratory) AE is elevated in the presence of sleep apnea, and model (c, ECG) AE indicates that arrhythmias contribute to its AE.

**Fig. 3:**
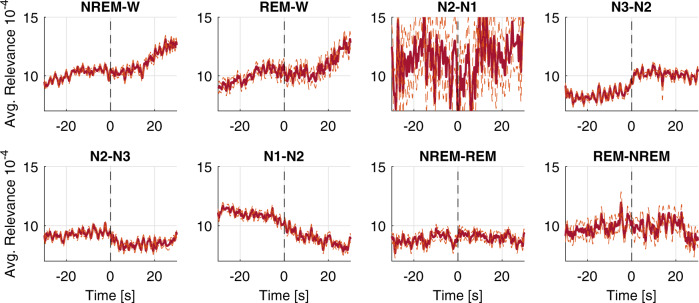
Average and smoothed relevance attribution averaged over channels of model (b; EEG+EOG+EMG) time-locked to sleep-stage transitions. The average relevance attribution time-locked to sleep-stage transitions. These were averaged in 4353 PSGs from the test set with available manual scoring. The dotted line marks the standard error of the mean. Relevance attribution was computed using gradient SHAP.

The relationship between relevance scores and manually scored sleep events was investigated to validate that these are meaningful to the AE models. Relevance scores were averaged around transitions of manually scored hypnograms, arousal, and apnea/hypopnea events in PSGs from the CFS, the MrOS, and SHHS cohort in the training set. In Fig. 3, relevance scores of model (b; EEG + EOG + EMG) time-locked to sleep-stage transitions are shown. On average, the relevance scores of model (b; EEG + EOG + EMG) are increased when transitioning to lighter sleep or wakefulness. Furthermore, as shown in Supplementary Fig. 2, the average relevance scores are affected by arousal and apnea.

### Association between age estimate error and mortality

Older age is the major predictor of mortality, an obvious application of our AEE calculation was to explore whether a positive AEE predicts increased mortality.

The combined dataset of subjects with both a PSG and associated mortality data consisted of 9386 subjects from the SHHS (*n* = 5696, deaths = 1285), the MrOS (*n* = 2781, deaths = 1662), and the WSC (*n* = 909, deaths = 98). This subset of data was also used in the training, validation, and test set for age estimation. The combined sample of subjects had a mean age of 66.0 ± 11.1 years at baseline and was followed for a median of 12.1 ± 3.7 years.

Supplementary Table 6 shows the association between all-cause mortality and a set of demographic, lifestyle, and health characteristics that we investigated with Cox proportional hazard models adjusted for age, sex, BMI, and cohort. The table also displays the proportion of missing data, which was imputed for further analyses.

The distributions of all demographics, lifestyle, and health characteristics across quartiles of the corrected AEE (AEEc, which is AEE corrected for age bias) for model (e, Ensemble–Avg.) are shown in Supplementary Tables 7–9 for the SHHS, WSC, and MrOS, respectively. Most notably, hypertension was more prevalent in the highest AEEc quartile.

After controlling for covariates (see Table 2), each 10-year increment in the AEE of model (e, Ensemble–Avg.), of which the standard deviation was 6.82 years in this combined dataset, was associated with a 29% (HR = 1.29, 95% confidence interval [CI]: 1.20–1.39) and 40% (HR = 1.40, 95% CI: 1.21–1.62) increase in all-cause and cardiovascular mortality rates, respectively. In Supplementary Tables 10–12, the results of the mortality analyses in each cohort are shown. Restricting the analyses to individual cohorts revealed that the association between AEE and mortality is present in the SHHS and MrOS cohort, while analysis in the WSC yielded mostly non-significant effects. However, this could be explained by a lower sample size and fewer deaths in the WSC (*n* = 909, deaths = 98) compared to the other cohorts.

**Table 2:** Mortality hazard ratios per 10-year increment in AEE in the combined data of the Sleep Heart Health Study, the Wisconsin Sleep Cohort, and the MrOS Sleep Study.

		Cox Model 1 HR (95% CI)	Cox Model 2 HR (95% CI)	Cox Model 3 HR (95% CI)
All-cause	(a) Central EEG	1.12 (1.07–1.17)	1.15 (1.10–1.20)	1.11 (1.06–1.16)
	(b) EEG+EOG+EMG	1.11 (1.06–1.17)	1.17 (1.11–1.24)	1.14 (1.08–1.20)
	(c) ECG	1.08 (1.04–1.12)	1.09 (1.06–1.13)	1.07 (1.03–1.11)
	(d) Respiratory	1.04 (1.00–1.09)	1.10 (1.04–1.16)	1.09 (1.03–1.15)
	(e) Ensemble–Avg.	1.23 (1.15–1.31)	1.38 (1.28–1.49)	1.29 (1.20–1.39)
Cardiovascular	(a) Central EEG	1.21 (1.11–1.32)	1.24 (1.14–1.36)	1.17 (1.07–1.28)
	(b) EEG+EOG+EMG	1.13 (1.03–1.25)	1.21 (1.09–1.34)	1.15 (1.04–1.28)
	(c) ECG	1.15 (1.08–1.22)	1.16 (1.09–1.24)	1.11 (1.04–1.19)
	(d) Respiratory	1.04 (0.95–1.13)	1.09 (0.97–1.22)	1.07 (0.96–1.19)
	(e) Ensemble–Avg.	1.36 (1.20–1.54)	1.58 (1.37–1.83)	1.40 (1.21–1.62)

The mortality analysis was performed with (*n* = 9386, deaths = 3045) for all-cause mortality and (*n* = 9188, death = 976) for cardiovascular mortality. *HR* hazard ratio, *AEE* age estimate error. Model 1: age. Model 2: age, sex, body mass index, race, smoking status, education level, daily alcohol intake, daily caffeine intake, benzodiazepines, sedatives, antidepressants, and cohort. Model 3: Model 2 + wake after sleep onset, N2%, REM%, arousal index, apnea-hypopnea index, sleep time with blood oxygen saturation below 80%, Epworth Sleepiness Scale Score, hypertension, congestive heart failure, history of heart attack, stroke, and type 2 diabetes.

**Fig. 4:**
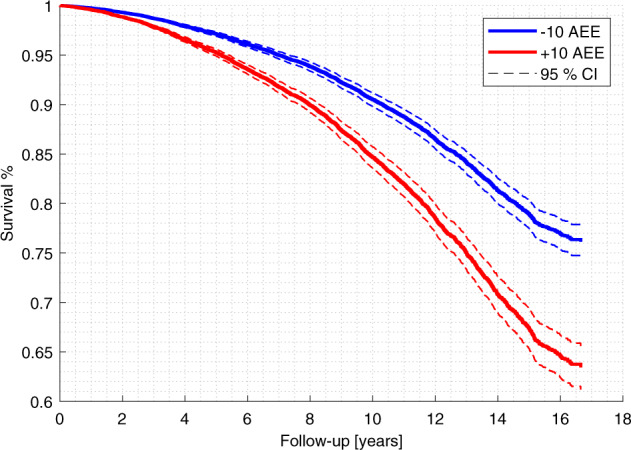
Survival curve for all-cause mortality with an AEE varying ±10 years. The survival curve was generated for all data (*n* = 9386, deaths = 3045) and model (e, Ensemble–Avg.) using the Cox proportional hazards model 3 shown in Supplementary Table 3. The 95% CI express the uncertainty in the modeled hazard ratio. AEE age estimate error, CI confidence interval.

In Fig. 4, survival curves for an AEE of +10 and −10 years for model (e, Ensemble–Avg.) is shown, which was generated using Cox Model 3 with all other covariates are set to their mean value. The survival curve was extended to compute the change in life expectancy for a change in AEE from −10 to 10 years. For model (e, Ensemble–Avg.), given an age of 40, 60, or 80 years in Cox Model 3, a decrease in life expectancy was 12.6 years (CI: 8.9–16.2), 8.7 years (CI: 6.1–11.4), or 6.0 years (CI: 4.2–7.8), respectively.

Because hypertension and sleep apnea were very common in these cohorts, we also examined the mortality association in subjects without hypertension and without sleep apnea (AHI ≥ 15). A sensitivity analysis (see Supplementary Table 13) found that isolating the analyses to a subset of subjects without hypertension (*n* = 5303, deaths = 1291) decreased the hazard ratios of increased AEE to (HR = 1.25, 95% CI: 1.11–1.40) and (HR = 1.31, 95% CI: 1.03–1.66) for all-cause and cardiovascular mortality, respectively. As shown in Supplementary Table 14, isolating the analyses to a subset of subjects without sleep apnea (*n* = 5161, deaths = 1390) decreased the hazard ratios of increased AEE to (HR = 1.22, 95% CI: 1.10–1.37) and (HR = 1.24, 95% CI: 1.01–1.54) for all-cause and cardiovascular mortality, respectively. These effects are significant within the 95% CI in both the hypertension and sleep apnea sensitivity analyses. Lastly, to justify the inclusion of training and validation data for our AE models, we restricted the analysis to the test set (*n* = 8432, deaths = 2601). As shown in Supplementary Table 15, the hazard ratios of AEE are slightly decreased to (HR = 1.27, 95% CI: 1.18–1.38) and (HR = 1.35, 95% CI: 1.16–1.56) for all-cause and cardiovascular mortality, respectively.

## Discussion

Our results show that deep learning enables precise age estimation and extraction of incipient and medically relevant information from PSGs that predict mortality beyond the capabilities of basic sleep metrics derived from sleep staging and apnea scoring. Subjects’ ages were estimated with an MAE of 5.8 ± 1.16 years with model (e, Ensemble–Avg.), while basic metrics had a MAE of 14.6 ± 5.91 years. We addressed the interpretability problem of deep learning methods using gradient SHAP, which suggested that the model’s estimates were largely driven by clinically known waveforms (e.g., sleep-stage transitions and apnea). We found that 10-year increments in AEE of the (e, Ensemble–Avg.) model was associated with increased all-cause mortality rate of 29% (HR = 1.29, 95% CI: 1.20–1.39) and increased cardiovascular mortality rate of 40% (HR = 1.40, 95% CI: 1.21–1.62). For a 60-year-old subject, the difference of −10 and +10 years in AEE translates to a decreased life expectancy of 8.7 years (CI: 6.1–11.4) for Cox Model 3, which adjusts for basic sleep metrics that are associated with early mortality.

The AE models performed well on the test set and generalized well to the HomePAP study test set with a MAE of 8.16 ± 3.75 years for the (e, Ensemble–Avg.) model. Based on these results, we expect the model to generalize to new data recorded in adult subjects aged 20–90 from other clinics, obtaining MAEs between 5.8 and 8.16 years. Calibrating the AE in new, unseen populations may however be necessary to achieve a MAE of 5.8 years in these instances. Further, although the model was trained using data that included children, this data was limited in amount and age range, so our model is not validated for use in children. We however note that validating similar age estimates in children in a separate study could have great interest for the study of neurodevelopmental disorders in children.

We found that model (e, Ensemble–Avg.) was biased for older subjects, estimating preferentially a younger age. This may be caused by either a regression to the mean of the predictions or by unhealthy subjects having died in the older (>80 years) population, i.e., a type of survival bias. Regression to the mean is a difficult issue to handle in non-linear models. The AE models output layer did not have any non-linear activation function, however, the AE still seem to have a non-linear clipping of AEs (e.g., around 83 years in Fig. 1). Given that the models have an uncertainty, it will drive the estimate away from the edge case of 90 years, on average this estimate would increase the loss. A similar effect is observed for young subjects in both the test set and HomePAP set, which exhibit systematic overestimation. It is likely that adjusting for this bias observed in the test set would improve performance in new data. Moreover, model (e, Ensemble–Avg.) had a significant (*p* = 2∙10^−9^) night-to-night variability, which may result from of the first-night effect. However, more PSGs with multiple nights are necessary to confirm this. Using multiple PSGs for age estimation may alleviate this problem.

A previous study used a linear model of sleep staging features based on EEG only to model “brain age” and reported a MAE of 7.6 years^27^. However, the results are difficult to compare as the dataset, age ranges, and investigated PSG signals differ.

The deep learning AE models appeared to largely rely on patterns that are known to be related to aging such as sleep fragmentation^24^. The relevance attribution analyses showed that transitions to deeper sleep would cause model (b; EEG + EOG + EMG) to estimate a lower age. The analysis of arousal and sleep apnea (Supplementary Fig. 2) showed that these modulate the AE. Relevance scores were computed using a baseline of zero, which affects how the relevance scores should be interpreted. For example, relevance scores were increased after but not during apnea/hypopnea events; however, this is expected as low amplitude breaths are likely healthier than the baseline of zero amplitude in complete apnea. Moreover, the gradient SHAP method assumes an independent and linear attribution from each sample to the AE^41,42^, which is not capable of accurately describing PSG patterns or the processing in the deep neural network. Therefore, we can only argue that the models probably use non-linear statistics related to these known patterns without strictly summarizing sleep patterns to the frequency of binarized events. Alternatively, we could have interpreted the model attention network weights, however, the long short-term memory networks render these weights difficult to interpret.

Survival analysis found that greater AEE was associated with increased all-cause and cardiovascular mortality. In the Cox Models for all AE models, AEEs had larger hazard ratios while controlling for demographics and medication than controlling for only age or including health and basic sleep metrics. We infer from this that (1) the AE is more meaningful when knowing demographics and medication, and (2) the AEE is not fully explained by basic sleep metrics such as sleep-stage distributions, ArI, and AHI. It is thus evident that a PSG contains much more information than what is summarized in basic sleep metrics. Our analysis (see Supplementary Table 5) of AEE in relation with morbidities found associations to T2D, hypertension, CHF, and history of heart attack, but pathways underlying these associations are difficult identify. Short sleep duration in insomnia has been shown to be associated with T2D^43^, which may explain the association in model (a, central EEG), and (b; EEG + EOG + EMG). Model (c, ECG) was associated with hypertension, CHF, and history of heart attack, which was expected as these factors affect the morphology of ECG. Hypertension was associated with increased AEE in all but model (d, Respiratory). A sensitivity analysis that excluded subjects with hypertension (see Supplementary Table 13) showed that AEE was still associated with increased mortality, although the effects were smaller. In future studies, the association between AEE and mortality risk should be investigated in completely unseen cohorts to study the generalizability of this effect.

A strength of this study is the inclusion of multiple cohorts, likely increasing generalizability of our models. This is however also a limitation as measuring sleep with a common instrumentation and in a more controlled environment could have better predictive power by reducing technical noise, first-night effect, variation in recording equipment, electrode placement, room temperature, and external noise, etc. Another limitation is that sleep varies from night-to-night and our AE relies on only one sleep study per subject. It is likely that multiple examinations per subject and establishing trajectories of aging would have stronger predictive power.

Other approaches for age estimation have relied on epigenetics^44^, proteomics^45^, neuroimaging^46,47^, etc., but few of these markers have been linked to hard outcomes such as mortality. Freire-Aradas et al. found that 7 DNA methylation markers estimated age with a median age error of ±3.07 years^44^. A systematic review of proteomic studies found that a 83-protein could estimate age with a MAE of 5.5 years^45^. Cole et al. leveraged T1-weighted magnetic resonance imaging (MRI) to estimate age with a MAE of 5.02 years. Moreover, each 1-year increment in this AE was associated with a 6.1% increased relative risk of all-cause mortality^47^. This corresponds to a hazard ratio of 1.29 for a 10-year increment, which is close to the hazard ratio we report in this study for the ensemble model. Advantages of PSG over these methods include being non-invasive, less expensive, and more accessible. It is also notable that sleep, should causality be demonstrated in future studies, can be modified by well-established behavioral and pharmacological therapies, unlike many of these other proxies.

In that sense, the AE may also serve as an outcome measure in adult subjects (20–90 years) for interventions in both clinical and research settings. Moreover, the AE could potentially serve as an easily understood marker of health for patients and the general public. In contrast, current sleep quality measures such as SE and N3% can be difficult to interpret for a given sleep clinic patient. Thereby, a sleep-based AEE could improve health literacy^48^ among patients. A recent Danish study found that interviews based on body age assessments motivates health promotion in the workplace, which lead to a decrease in smoking and metabolic syndrome among the employees^49^. Moreover, a meta-analysis found that health literacy was correlated with treatment adherence, especially among vulnerable groups^50^. These findings may apply to sleep health as well, which could lead to better adherence to treatment such as lifestyle changes and continuous positive airway pressure (CPAP) therapy. This could be interesting to investigate in future studies.

Finally, our predictions of mortality are estimated through cardiovascular, respiratory, and brain activity related to aging, which we hypothesize is a likely proxy of premature aging, but not likely the sole or even main predictor of mortality. This is illustrated by the fact we recently found that reduced REM sleep amounts also significantly predicted mortality in these same samples^13^, and as shown in Cox Model 3 in Supplementary Table 3 that adjust for REM sleep%, addition of REM sleep to the Cox Model did not diminish predictive effect of AEE on mortality. Clearly, other factors than AEE in the PSG are likely incipient biomarkers of poor health predicting mortality or new-onset morbidity. Additional approaches aiming at directly predicting mortality^51^ and the development of cardiovascular and brain morbidity in these cohorts with and without controlling for AEE may help to uncover additional information in PSG recordings.

## Methods

### Data description

Diversity of data is a necessity for the success of a supervised deep-learning algorithm^52^. Olesen et al. showed that both data quantity and diversity were essential for automatic sleep staging, a supervised learning task, using polysomnography data^53^. Diversity of data can be ensured using polysomnography recordings from multiple study cohorts with different study objectives and patient populations.

In this study, we included participants with a wide age range from seven study cohorts: STAGES^31,32^, the SSC^33,34^, the WSC^4,34^, the SHHS^35,36^, the MrOS^36–38^, the CFS^36,39^, and HomePAP Study^36,40^. Access to the SHHS, MrOS, CFS, and HomePAP Study was granted through the National Sleep Research Resource^36^. This study was approved by institutional review boards and written informed consent was obtained from all participants. The included study cohorts are briefly described in the subsections below:

#### The stanford technology analytics and genomics of sleep

The STAGES^31,32^ is a prospective cross-sectional multi-site cohort designed to investigate the relationship between different sleep-related data including in-lab polysomnography, questionnaire data, genomics, actigraphy data etc. A total of 1859 PSGs were recorded in 1627 participants of ages between 13 and 83 at the following 6 clinical sites: Stanford University, Bogan Sleep Consultants, Geisinger Health, Mayo Clinic, MedSleep, and St. Luke’s Hospital. A total of 1536 PSGs in 1494 participants were included, while the remaining PSGs were excluded for being a split-night study or due to missing annotations. The study was approved by institutional review boards at each site.

#### The Wisconsin sleep cohort

The WSC^4,34^ is an ongoing longitudinal population-based cohort of employees from Wisconsin state agencies, and it approximates a population-based sample, although they are generally more overweight^4^. A total of 1682 PSGs in 962 participants was included, which aged between 37 and 78. The participants were tracked through 2018 and deaths were identified by matching social security numbers with death record sources^13^. A detailed description of the cohort can be found in Young et al.^4^ and Moore et al.^34^. Cardiovascular mortality was categorized using the same rules as Leary et al.^13^. The study has been reviewed and approved by the University of Wisconsin Institutional Review Board.

#### The Stanford sleep cohort

The SSC^33,34^ is a cohort of patients who underwent in-lab PSG at the Stanford Sleep Clinic. A total of 700 independent PSGs was included in patients aged between 13 and 90. A detailed description of the cohort can be found in Andlauer et al.^33^.

#### The MrOS sleep study

The MrOS Sleep Study^36–38^ is a multi-site cohort of older men to study the association between sleep disorders and vascular disease, falls, fractures, and mortality. A total of 2874 male participants were included who underwent full in-home PSG recording. Vital status was determined based on contact every 4 months, or in case of no response, by their next-of kin. Reported deaths were confirmed by centralized review of death certificates^13,37^. Deaths through August 2018 were included in these analyses. Cardiovascular mortality was categorized using the same rules as Leary et al.^13^. The study was approved by the institutional review board at each of the six sites: University of Alabama at Birmingham, University of Minnesota, Stanford University, University of Pittsburgh, Oregon Health and Science University, and University of California, San Diego.

#### The Cleveland family study

The CFS^36,39^ is a large family-based study of sleep apnea, consisting of probands with sleep apnea, neighborhood controls, and their family members. We included PSG recordings obtained in the Clinical Research Center from 730 participants of age between 6 and 88 years. The study was approved by the institutional review committee at the University Hospitals Case Medical Center.

#### The sleep heart health study

The SHHS^35,36^ is a large multi-center cohort designed to study the association between sleep apnea and cardiovascular disease. We included 5703 participants, aged between 40 and 90, were studied with in-home PSG. Participants vital status was continually identified and confirmed using interviews, written annual questionnaires, contact to next-of-kin, hospital records, obituaries, and linkage with the Social Security Administration Death Master File^35,54^. Cardiovascular mortality was categorized as recorded by parent studies^35,54^. The study was approved by institutional review boards at each of the six sites: University of Arizona, Boston University, University of California-Davis, Johns Hopkins University, University of Minnesota, and New York University.

#### The home positive airway pressure study

The HomePAP Study^36,40^ is a multi-site randomized controlled trial with the aim of comparison in-lab PSG and in-home unattended portable monitoring for diagnosis of obstructive sleep apnea and CPAP treatment. We included 190 patients of age between 20 and 80 with in-lab PSG without full or split-night CPAP at one of 7 sites: Case Western Reserve University affiliates (University Hospitals, MetroHealth Medical Center, and Cleveland Clinic), Northwestern University, University of Wisconsin in Madison, University of Minnesota, and University of Washington. The study was approved by institutional review boards at each site.

#### Data use and study design

Across the cohorts, PSGs were excluded if the participant’s age was unknown, the recording was a CPAP split-night, the recording included <3 h of sleep, or if more than one of the PSG signals were missing.

**Fig. 5:**
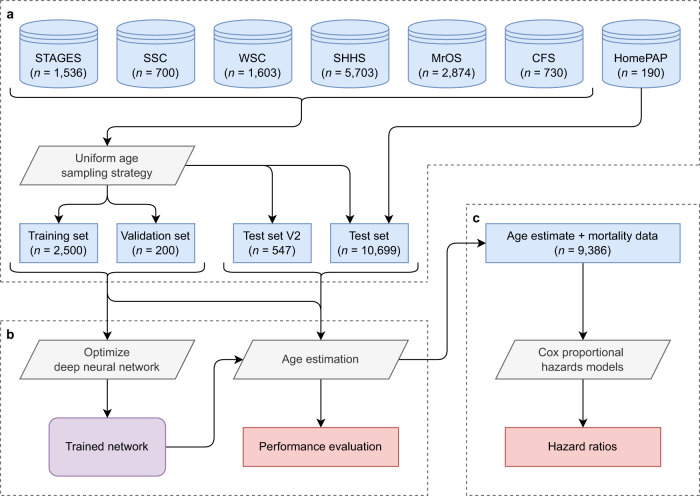
Use of data for age estimation and evaluating mortality risk. **a** The data from six cohorts are sampled to generate a training and validation set with a uniform age distribution. The remaining data comprises a test set, some of which has additional visits (test set V2). **b** Age estimation models are optimized and evaluated in all data. **c** Associations between increased age estimate errors and mortality risk are evaluated in all available data using Cox proportional hazards models.

To facilitate the development and testing of the AE models, the combined data were split into a training set (*n* = 2500), a validation set (*n* = 200), a test set (*n* = 10,699), and a second test set comprised of repeat visits (*n* = 547). The AE models are developed using the training and validation set, which should include diverse data at all ages. To ensure this, we propose a sampling strategy with uniform age distribution in favor of the commonly used random sampling. Firstly, patients who used CPAP or had any known neurological disorders including narcolepsy and RBD were allocated to the test set. Data was sampled for the training set by iteratively excluding data with the most represented age, cohort, and sex, see Supplementary Table 16 for details. Similarly, the validation set uses the same algorithm with the remaining data. The high-level flow of data from each cohort to various sets used for age estimation and evaluation of mortality risk is shown in Fig. 5.

The test set used the remaining data, which was not uniform but can be analyzed stratified by age. Participants with an age >89 were recorded as being 90, therefore we chose to exclude these from the test set. Moreover, data from the HomePAP Study (*n* = 190) was left out of the remaining test set for an additional test set, which provided an unbiased performance estimate as no data from the cohort is included in the training or validation set. Supplementary Fig. 3 shows the distribution of age and cohorts across the training, validation, and test sets. Supplementary Table 17 shows the distribution of basic PSG metrics across the included cohorts. The apneas and hypopneas were scored in agreement with AASM guidelines^1^, which requires associations with either a 3% desaturation or an arousal for hypopneas. Arousals were either scored manually in agreement with the AASM guidelines^1^ (CFS, MrOS, SHHS, HomePAP) or automatically scored using a previously validated method^21^ (STAGES, WSC, SSC) when manual annotations were missing.

### Preprocessing of polysomnographic signals

The PSG data included in this study have been recorded at many clinical sites with varying signal montages, environments, technicians, equipment, software, and acquisition settings. These differences are addressed in the preprocessing step to both standardize the data and eliminate signal artifacts. Specifically, we implemented a preprocessing that can (1) select the appropriate signal derivations; (2) resample signals to a desired and standardized sampling frequency; (3) eliminate signal artifacts; and (4) normalize signal amplitudes.

A PSG recording involves measuring many physiological signals and these can vary between recordings. Most commonly, the PSG recording includes electroencephalography (EEG) signals, electrooculography (EOG) signals (left and right), electromyography (chin), electrocardiography (ECG), nasal pressure, oral airflow, plethysmography belts (abdominal and chest), and blood oxygen saturation. Except for frontal and occipital EEG, we included all of these as they were available in almost all cohorts. Potential missing signals was substituted for flat signals of zeros.

The convolution neural networks (CNNs) assume a constant sampling rate, therefore, the signals are resampled to a sampling frequency of 128 Hz, which enables all signals to be stacked in one tensor. This frequency was chosen as it preserves most relevant information while still imposing a relatively low computational burden. The resampling was implemented with a finite impulse response (FIR) low-pass filter with a Kaiser window. However, the blood oxygen saturation was resampled with linear interpolation.

Thereafter, signals were filtered using infinite impulse response (IIR) filters to eliminate artifacts and ensure that signals contained similar spectral content across recordings. The IIR filters were implemented as elliptic filters with an order of 16, a maximum passband ripple of 1 dB, and minimum stopband attenuation of 40 dB. The cut-off frequencies for the filters were the following: EEG and EOG: band-pass (0.3–45 Hz); EMG: high-pass (10 Hz); ECG: high-pass (0.3 Hz); nasal pressure: high-pass (0.1 Hz); airflow and plethysmography belts: band-pass (0.1–15 Hz); and blood oxygen saturation: no filtering. All filters were applied forwards and backwards to avoid signal phase distortion.

Finally, the signal amplitudes, except for the blood oxygen saturation, were normalized such that −1 and 1 corresponded to the 5th and 95th percentiles. Although, the blood oxygen saturation was normalized such that −1 and 1 corresponded to 60% and 100% saturation. The normalization enables efficient training of neural networks^52^.

### Deep learning framework for age estimation models

The proposed deep learning framework for AE was designed to input *C* number of preprocessed PSG signals of *T* samples ${{{\boldsymbol{x}}}} \in {\mathbb R}^{C \times T}$ and output an estimate of the subject’s age $\hat y \in {\mathbb R}_ +$. In the subsections below, the network architecture, the optimization approach, performance testing, and the interpretation of models is presented.

A challenge in end-to-end deep learning processing of PSG recordings is the huge data size, which usually is of 8 h corresponding to an input dimension of ${{{\boldsymbol{x}}}} \in {\mathbb R}^{12 \times (128 \times 60 \times 60 \times 8)}$ corresponding to roughly 177 MB in 32-bit float. Directly optimizing a network to map the whole night’s PSG to estimate age is practically infeasible as intermediate network activations must be saved for optimization through backpropagation. Therefore, we chose to optimize the networks in two phases.

Phase (1): Estimating age based on 5-min epochs of PSG data ${{{\boldsymbol{x}}}}_{{{\boldsymbol{i}}}} \in {\mathbb R}^{C \times (128 \times 60 \times 5)}$.

Phase (2): Estimating age based on the latent space learned ${{{\boldsymbol{z}}}}_{{{\boldsymbol{i}}}} \in {\mathbb R}^M$ in phase 1 (at the network layer preceding the output layer) for all 5-min epochs of length *q* in a whole night’s recording ${{{\boldsymbol{z}}}} \in {\mathbb R}^{M \times \left\lfloor {T/q} \right\rfloor }$.

Thereby, the networks first learn signal patterns in 5-min epochs that are associated with aging, and secondly, distributions of these patterns across the night taken into consideration by the networks.

#### Neural network architecture of age estimation models

The network incorporates a series of structures that have shown success in sleep-stage classification from PSG data^19,53,55,56^, image classification^57^, and natural language processing^58^.

As shown in phase (1) in Supplementary Fig. 4, 5-min epochs of data ***x***_***i***_ are processed through a channel mixing layer, a CNN using inverted residual bottleneck blocks (see Supplementary Fig. 5), a bi-directional long short-term memory^59^ (Bi-LSTM) layer, an additive attention^58,60^ layer, and two dense layers, which produces an estimate of age $\hat y_{P1_i}$.

As shown in phase (2) in Supplementary Fig. 4, the latent space ***z***_***i***_ is concatenated from the layer activation in phase (1) at the last layer and the average activation after the Bi-LSTM layer to summarize the 5-min epochs of data ***x***_***i***_. Like phase (1), the whole night’s latent space ***z*** is processed through a Bi-LSTM layer, an additive attention layer, and two dense layers, which produce a final AE $\hat y_{P2}$.

The implementation details of each neural network type^61–65^ are presented in the Supplementary Notes.

#### Optimization scheme for age estimation models

The network was optimized in two phases as outlined in Supplementary Fig. 4 to both lower the computational burden and increase the amount of training data. The Huber loss used as the objective function to minimize and was defined as

1 $$L_H = f\left( x \right) = \left\{ {\begin{array}{*{20}{l}} {\frac{1}{2}\left( {y - \hat y} \right)^2,} \hfill & {{{{\mathrm{for}}}}\left| {y - \hat y} \right| \,<\, 5} \hfill \\ {5\left( {\left| {y - \hat y} \right| - \frac{1}{2}5} \right),} \hfill & {{{{\mathrm{otherwise}}}},} \hfill \end{array}} \right.$$

which corresponds to an L2 loss for an error <5 years and L1 loss otherwise. This loss weighs outliers less than an L2 loss while retaining a continuous gradient. The loss was further divided by a factor of 112.5 such that an error of 25 years corresponds to a loss of 1. Using this loss and L2 weight decay (not counting network bias’s), the network was optimized using Adam optimization^66^ with *β*_1_ = 0.9 and *β*_2_ = 0.999.

Additional optimization settings and hyperparameter tuning methods^67^ are described in Supplementary Notes.

We experimented with various combination of PSG signals: (a, Central EEG) C3-A2, C4-A1; (b, EEG+EOG+EMG) C3-A2, C4-A1, L-EOG, R-EOG, chin EMG; (c, ECG) ECG; (d, Respiratory) airflow, nasal pressure, thoracic and abdominal belts, SaO2.

Moreover, an ensemble model (e, Ensemble–Avg.) was developed based on models (a), (b), (c), and (d).

Finally, as a comparison to basic sleep summary measures, a linear regression model using sex, BMI, ArI, AHI, TST, WASO, and percentage of N1, N2, N3, and REM sleep was developed.

#### Performance quantification of age estimation models

The performance of the AE was quantified using mean absolute error (MAE) and Pearson’s correlation coefficient. The test set was not characterized by a uniform age distribution; therefore, we measured the MAE stratified by 5-year age intervals (MAE_i_) with intervals ([20, 25], [25, 30], …, [85–89]). The average MAE across age intervals MAE_i_ was used as a final measure of performance.

#### Interpretation of age estimation models

Deep neural networks are traditionally considered black boxes due to their complexity, which is of high concern generally and even more so in a clinical setting. However, in recent years several methods have been proposed that can interpret network decisions in a meaningful way^68,69^. We applied gradient SHAP^41,42^ to distribute relevance scores to each PSG sample using phase (1) of the optimized networks. To remove noise, the sample relevance scores were filtered by a Gaussian window with a length of 10 s and standard deviation of 0.234 s. Relevance scores were averaged around transitions of manually scored hypnograms, arousal, and apnea/hypopnea events in PSGs from the CFS, MrOS, and SHHS cohort in the test set. Here we expected to see increases in relevance scores arousals, transitions to lighter sleep, and sleep apnea.

Moreover, we examined statistical associations between the AEE and conventional sleep parameters from manual scoring.

### Association between age estimation and mortality

The usefulness of the AEE as a marker for sleep health was examined by studying its association with all-cause mortality. This analysis was performed in the SHHS, MrOS, and WSC.

#### Statistical analyses

We considered that missing data were missing at random and these were imputed using multivariate imputation by chained operationalized equations using R 4.0.4 MICE package^70^. Information about CPAP had a lot of missing data (0 for WSC, 5603 for SHHS, and 2671 for MrOS), therefore, we excluded the few subjects (*n* = 74) that used CPAP from these analyses.

We employed Cox proportional hazards models to evaluate associations between AEE and all-cause mortality. The results are reported as hazard ratios (HR) along with their 95% confidence intervals (CI) for every 10 years increase in AEE, which is close to the standard deviation of AEE.

The Cox proportional hazards models controlled for a combination of variables based on clinical and empirical knowledge^13^. Covariates were included in three combinations to investigate if the association was dependent on these covariates. Cox model 1 adjusted for age; Cox model 2 that included covariates we clinically known or suspect to influence mortality: age, sex, BMI, race, education, smoking status, daily alcohol intake, daily caffeine intake, medication use (antidepressants, benzodiazepines, and sedatives), and study site. Cox model 3 that included covariates from Cox model 2 and covariates empirically found to affect mortality in the MrOS cohort using 6-fold cross validation^13^: NREM 2%, REM%, SaO2-80, WASO, ArI, ESS, congestive heart failure, chronic obstructive pulmonary disease, type 2 diabetes, heart attack, and stroke. The proportional hazards assumption for AEE was confirmed graphically by analyzing the scaled Schoenfeld residuals.

A summary of all covariates was computed for each quartile of the AEE corrected for age variation (AEEc) for model (h, Ensemble–Avg. EEG). The AEEc was computed as the residuals in the linear regression model AEE = 1 + age + *ε*, i.e., where *ε* is the AEEc.

Fitted Cox proportional hazards models were formulated as survival functions $S_0^{KM}\left( t \right)^{z(x)}$, where $S_0^{KM}\left( t \right)$ is the baseline survival function and $z\left( x \right) = \exp (\beta _0 + \beta _1x_1 + \ldots + \beta _nx_n)$. The survival functions were plotted with the AEE as ±10 years, corresponding to the estimated hazard ratio exp (*β*_*AEE*_ × 10). Moreover, similar to a previous approach^28^, we computed the effect of an increased AEE on life expectancy by extending the survival curve and computing the difference in curve area. The baseline survival curves were extended by fitting a Weibull distribution $S_0^W(t)$. Life expectancy was computed as the area of $S_0^W\left( t \right)^{z(x)}$ with age set to 40, 60, or 80 years and the other covariates to their median in that age range ±10 years. The difference in life expectancy was found by subtracting the LE for *AEE* = −10 and *AEE* = 10.

### Reporting summary

Further information on research design is available in the Nature Research Reporting Summary linked to this article.

## Supplementary information

Supplementary Information

Nature Reporting Summary

## Data Availability

Polysomnography data included in this study was subject to data sharing agreement but is available upon reasonable request from E.M. (for STAGES and SSC), P.E.P (WSC), or upon request from the NSRR^36^ (SHHS, MrOS, CFS, and HomePAP).

## Code availability

The AE models were implemented, trained, and tested using PyTorch^71^ v. 1.7.1. All source code is available at https://github.com/abrinkk/psg-age-estimation, which includes instructions for use.
