# Supplementary material for: Age estimation from sleep studies using deep learning predicts life expectancy
Source: NPJ Digit Med. 2022 Jul 22;5:103. doi: 10.1038/s41746-022-00630-9 (PMC9307657; doi:10.1038/s41746-022-00630-9)
Supplement: Supplementary file 1 — Supplementary Information [file 41746_2022_630_MOESM1_ESM.docx]

**Supplementary Information**

**Age Estimation from Sleep Studies using Deep Learning Predicts Life Expectancy**

Andreas Brink-Kjaer^a,b,c^, Eileen B. Leary^c^, Haoqi Sun^d^, M. Brandon Westover^d^, Katie L. Stone^e,f^, Paul E. Peppard^g^, Nancy E. Lane^h^, Peggy M. Cawthon^e,f^, Susan Redline^i,j^, Poul Jennum^b*^, Helge B.D. Sorensen^a*^, Emmanuel Mignot^c*^

*Shared last author

^a^ Department of Health Technology, Technical University of Denmark, Kongens Lyngby, Denmark

^b^ Danish Center for Sleep Medicine, Department of Clinical Neurophysiology, Rigshospitalet, Denmark

^c^ Stanford Center for Sleep Sciences and Medicine, Stanford University, Palo Alto, CA, USA

^d^ Department of Neurology, Massachusetts General Hospital, Boston, MA, USA

^e^ Research Institute, California Pacific Medical Center, San Francisco, CA, USA

^f^ Department of Epidemiology and Biostatistics, University of California, San Francisco, CA, USA

^g^ Department of Population Health Sciences, University of Wisconsin-Madison, Madison, WI, USA

^h^ Department of Medicine, University of Davis School of Medicine, Sacramento, CA, USA

^i^ Department of Medicine, Harvard Medical School, Boston, MA, USA.

^j^ Department of Medicine, Brigham and Women’s Hospital, Boston, MA, USA.

Corresponding author: Emmanuel Mignot

Stanford Center for Sleep Sciences and Medicine, Stanford University, Palo Alto, CA, USA

E-mail: mignot@stanford.edu

**Supplementary Notes**

**Neural network architecture of age estimation models**

***1 Channel Mixing***

The preprocessed data in 5-minute epochs are input to a channel mixing layer, which is implemented as a 2D CNN with a $C\times1$ kernel and 32 features maps followed by batch normalization^1^ and rectified linear unit 6 (ReLU6) activations. This approach enables nonlinear channel mixing and is adapted from recent methods for automatic sleep stage classification and sleep event detection^2–4^.

***2 Feature Extraction with Convolutional Neural Networks***

The proposed network includes a CNN inspired by MobileNetV2^5^, which employs inverted residual bottleneck blocks to lower the computation burden while retaining high performance^5,6^. We employed a variation, as shown in Supplementary Fig. 5, of their architecture to further lower the computational burden. This variation uses convolutions in the time dimension and outputs a set of features with dimension $\mathbb{R}^{128\times1\times(q/320)}$.

***3 Temporal Analysis with Bi-Directional Long-short term memory networks***

Temporal dependencies are incorporated in the features using Bi-LSTM networks, which allows features to indirectly model concepts such as arousals and cyclic alternating patterns^7^. In phase (1) of the network, the Bi-LSTM outputs 128 features in both forward and backward LSTM layers. In phase (2) of the network, the Bi-LSTM has an outputs size of $32\times n_{size-P2}$ in both forward and backward LSTM layers, where $n_{P2}$ is a hyperparameter subject to optimization. Moreover, the number of Bi-LSTM layers $n_{LSTM-P2}$ in phase (2) of the network is additionally subject to optimization.

***4 Summarization with Additive Attention***

The concatenated forward and backward features from the Bi-LSTM are summarized along the temporal dimension using additive attention^8,9^ defined as

$$\begin{aligned} \boldsymbol{u}_{t}=\tanh(\boldsymbol{W}_{h}\boldsymbol{h}_{t}+\boldsymbol{b}_{t}) \#\left( 1 \right) \end{aligned}$$

$$\begin{aligned} \alpha_{t}=\frac{\exp\left( \boldsymbol{W}_{u}\boldsymbol{u}_{t} \right)}{\sum_{i=1}^{N} \exp\left( \boldsymbol{W}_{u}\boldsymbol{u}_{i} \right)}\#\left( 2 \right) \end{aligned}$$

$$\begin{aligned} \boldsymbol{c}=\sum_{t=1}^{N} \alpha_{t}\boldsymbol{h}_{t}, \boldsymbol{\#}\left( 3 \right) \end{aligned}$$

where $\boldsymbol{h}_{t}$ is the input features at time $t$, $(\boldsymbol{W}_{h}, \boldsymbol{W}_{u}, \boldsymbol{b}_{t})$ are network parameters, and $\boldsymbol{c}$ is a context vector. The additive attention layer uses an intermediate product $\boldsymbol{u}_{t}$ with a size of 512. Additive attention allows the network to focus on time periods of interest.

***5 Estimation with Dense networks***

Dense layers input the context vector $\boldsymbol{c}$ in both phase (1) and (2) to provide the AE. The first dense layer has a size of 256 and $64\times n_{P2}$ in phase (1) and (2) of the network, respectively. The first dense layer in each phase is followed by a ReLU activation and a dropout layer^10^.

**Optimization settings and hyperparameter tuning**

Usually in deep neural networks, network biases are initialized at zero^11^. However, since our training age range spans from 6 to 90 years, we found that setting the output bias to 50 would greatly decrease the required number of training epochs.

Early stopping was implemented such that training was interrupted if the validation loss had not decreased in three epochs.

In phase (1), the network was optimized for at most 20 epochs using a learning rate of $5\times{10}^{-4}$, a L2 weight decay penalty factor of ${10}^{-4}$, a dropout probability before last dense layer of 0.75, and a batch size of 32. The included PSG data could have missing channels, which were not missing at random across cohorts. To mitigate this, dropout was also applied to each input signal with a dropout probability of 0.1.

In phase (2), the network was optimized for at most 200 epochs with a batch size of 64 and with remaining hyperparameters tuned by Bayesian optimization^12^. Specifically, the following hyperparameters were explored in these ranges: learning rate $\in[{10}^{-6}, {10}^{-2}]$, L2 weight decay penalty factor $\in[{10}^{-8}, {10}^{-3}]$, dropout probability before last layer $\in[0, 0.99]$, network size $n_{size-P2}\in[1, 10]$, and number of Bi-LSTM layers $n_{LSTM-P2}\in[1, 3]$. The search space was initially explored in quasi-random Sobol sequence of 10 points followed by 20 experiments with a Gaussian Process model to minimize the final validation loss. The Gaussian Process model was implemented with expected improvement as an acquisition function.

**Supplementary Figures**

**Supplementary Fig. 1: Examples of interpretation through relevance attribution of samples.**


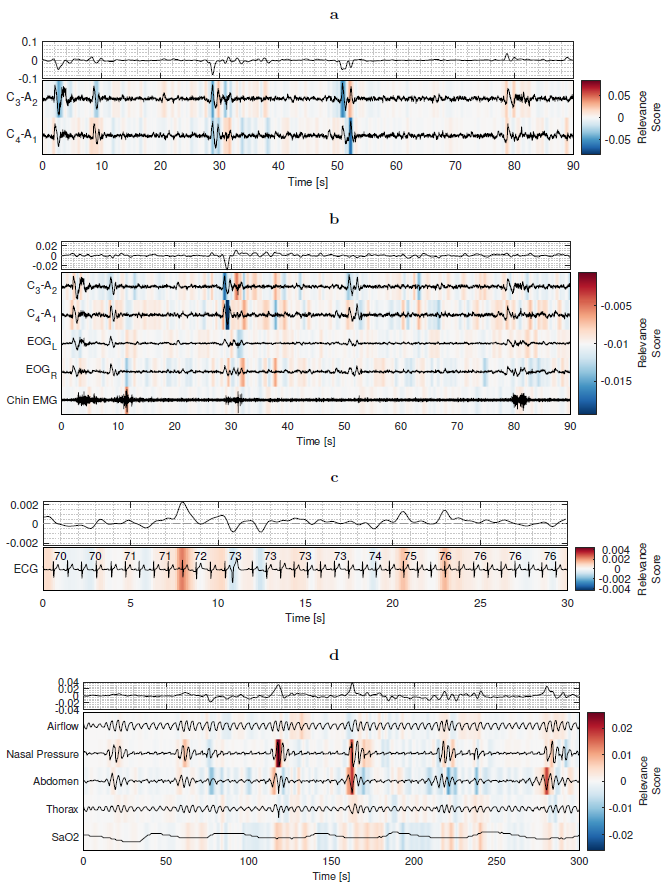


Relevance attribution averaged across channels is shown on top. Relevance attribution was computed using gradient SHAP.

**Supplementary Fig. 2: Average and smoothed relevance attribution averaged over signals time-locked to sleep events.**


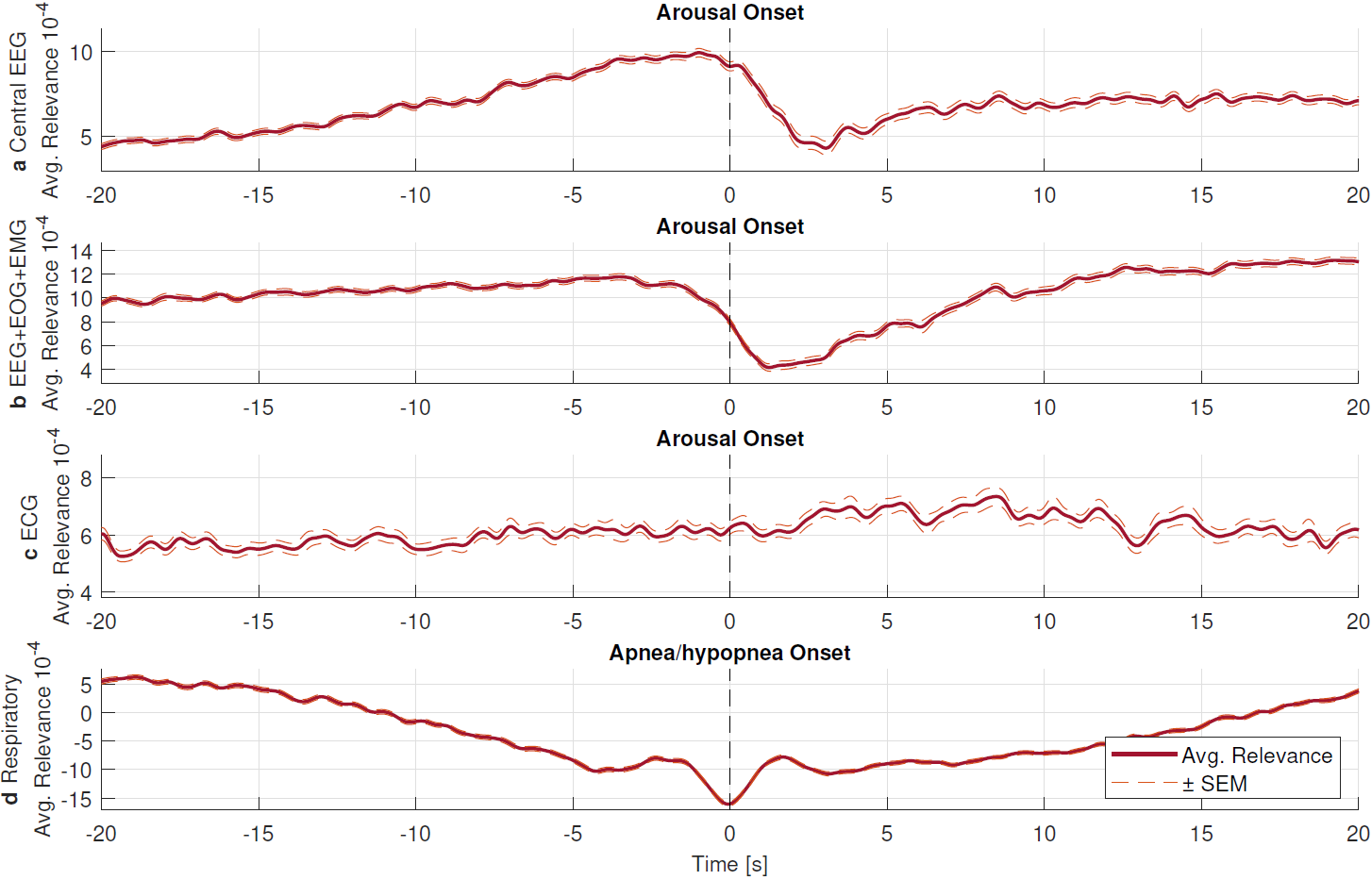


The average relevance attribution time-locked to sleep events. These were averaged in n = 5,036 PSGs from the test set with available manual scoring. The dotted line marks the standard error of the mean. Relevance attribution was computed using gradient SHAP.

**Supplementary Fig. 3. Stacked bar plots of data sets for developing and testing the age estimation models.**


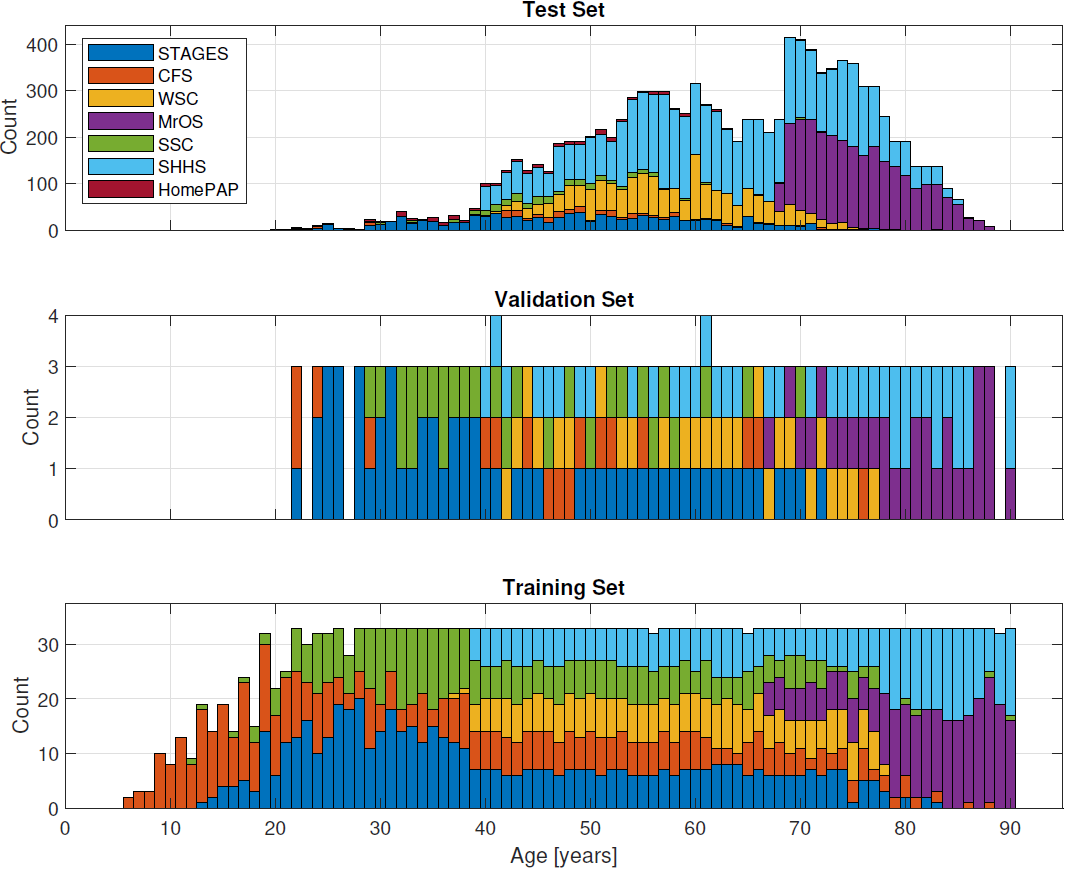


The data was split into a training set (*n* = 2,500), a validation set (*n* = 200), and a test set (*n* = 11,471). The age distributions were sampled using the algorithm in Supplementary Table 16. CFS: Cleveland Family Study; STAGES: Stanford Technology Analytics and Genomics of Sleep; WSC: Wisconsin Sleep Cohort; MrOS: MrOS Sleep Study; SSC: Stanford Sleep Cohort; SHHS; Sleep Heart Health Study; HomePAP: Home Positive Airway Pressure Study.

**Supplementary Fig. 4: Overview of age estimation framework.**
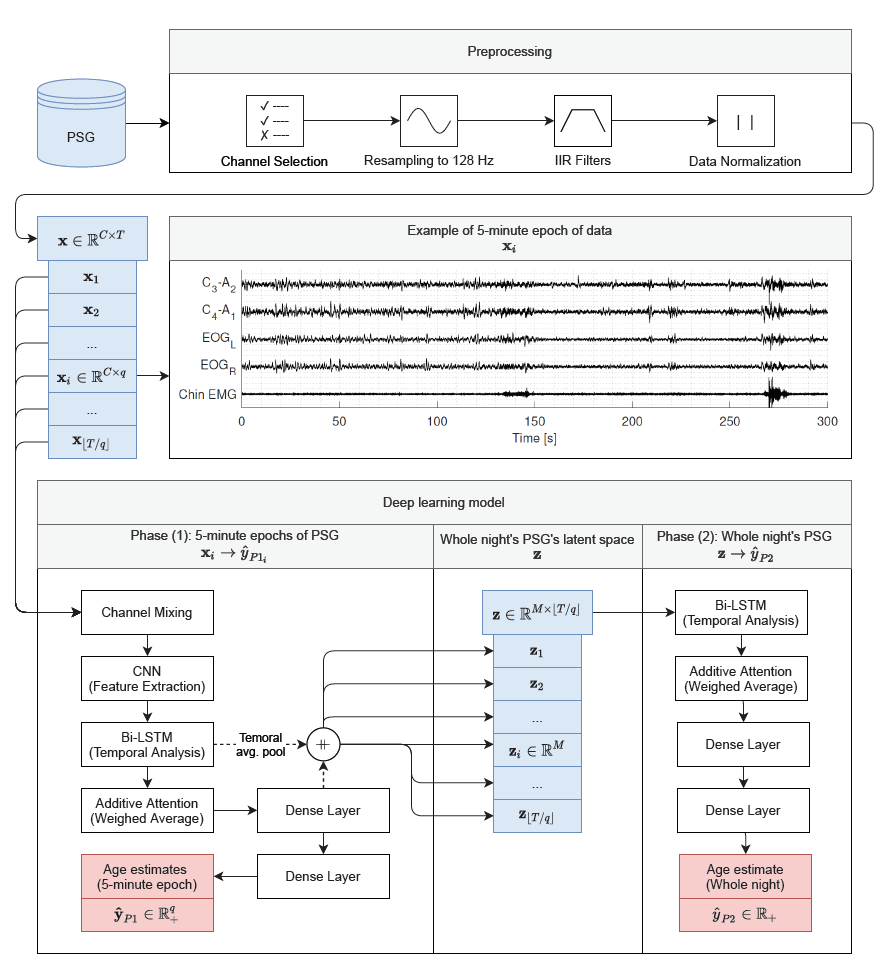


In the age estimation framework, the PSG $\boldsymbol{x}$ is preprocessed, split into epochs of 5 minutes, processed through a deep neural network in two phases. In phase (1), each epoch $\boldsymbol{x}_{\boldsymbol{i}}$ is input to a network that estimates age ${\hat{y}_{P1}}_{i}$ and saves a latent space $\boldsymbol{z}_{i}$. In phase (2), the latent space $\boldsymbol{z}$ for all epochs is processed to provide a global estimate of age $\hat{y}_{P2}$. PSG: polysomnography, IIR: infinite impulse response, C: number of PSG signals, T: total time of PSG, q: time in a PSG epoch (5 minutes), CNN: convolutional neural network, Bi-LSTM: bidirectional long short-term memory neural network, M: size of latent space for each PSG epoch.

**Supplementary Fig. 5: Convolutional neural network for age estimation.**


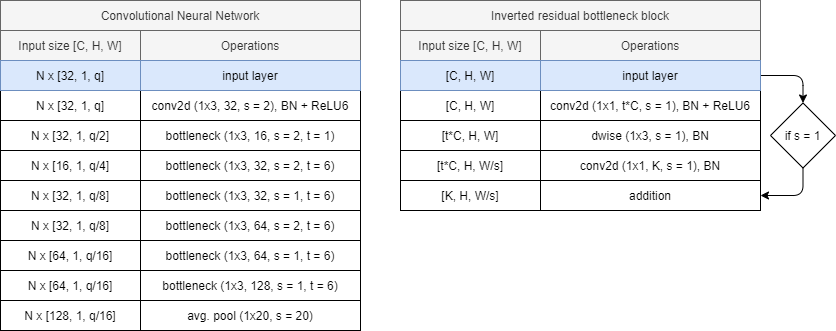


The convolutional neural network uses a series of inverted residual bottleneck blocks followed by a temporal average pooling. C: channel size, H: height, W: width, N: batch size, BN: batch normalization, ReLU6 Rectified Linear Unit 6: $f\left( x \right)=\min\left( \max\left( x,0 \right),6 \right)$, s = stride, t = expansion rate, K = output filter size.

**Supplementary Tables**

**Supplementary Table 1: Results of Bayesian optimization of hyperparameters.**

|  | Search space | (a) Central EEG | (b) EEG+EOG+EMG | (c) ECG | (d) Respiratory |
| --- | --- | --- | --- | --- | --- |
| Learning rate | $\mathbb{R}\in[{10}^{-6}, {10}^{-2}]$ | ${3.3\cdot10}^{-3}$ | $8.4\cdot{10}^{-5}$ | $6.2\cdot{10}^{-4}$ | ${7.8\cdot10}^{-3}$ |
| L2 weight decay penalty factor | $\mathbb{R}\in[{10}^{-8}, {10}^{-3}]$ | ${1.2\cdot10}^{-5}$ | ${9.1\cdot10}^{-7}$ | ${6.1\cdot10}^{-4}$ | ${6.6\cdot10}^{-6}$ |
| Dropout probability before last layer | $\mathbb{R}\in[0, 0.99]$ | $0.392$ | $0.271$ | $0.353$ | $0.282$ |
| Network size ($\boldsymbol{n}_{\boldsymbol{size-P}\boldsymbol{2}}$) | $\mathbb{N}\in[1, 10]$ | $7$ | $6$ | $5$ | $6$ |
| Number of Bi-LSTM layers ($\boldsymbol{n}_{\boldsymbol{LSTM-P}\boldsymbol{2}}$) | $\mathbb{N}\in[1, 3]$ | $2$ | $2$ | $3$ | $2$ |

Optimal hyperparameters used to design and optimize phase (2) of the age estimation models.

**Supplementary Table 2: Mean absolute error of test set stratified by 5-year age intervals.**

| Age Range [years] | Basic sleep measures | (a) Central EEG | (b) EEG+EOG+EMG | (c) ECG | (d) Respiratory | (e) Ensemble – Avg. |
| --- | --- | --- | --- | --- | --- | --- |
| [20 - 25]  *n* = 13 | 19.7 ± 6.82 | 4.25 ± 2.73 | 6.06 ± 4.45 | 12.5 ± 7.74 | 7.89 ± 5.06 | 6.31 ± 3.65 |
| [25 - 30]  *n* = 36 | 17.1 ± 6.99 | 5.65 ± 3.97 | 6.64 ± 7.45 | 13.9 ± 10.4 | 7.49 ± 4.79 | 7.18 ± 4.47 |
| [30 - 35]  *n* = 100 | 15.9 ± 7.56 | 3.81 ± 4.16 | 5.25 ± 5.85 | 11 ± 11.5 | 6.62 ± 5.42 | 5 ± 4.74 |
| [35 - 40]  *n* = 105 | 12.2 ± 9.21 | 5.29 ± 5.74 | 6.17 ± 4.95 | 8.46 ± 8.2 | 6.36 ± 4.97 | 4.03 ± 3.41 |
| [40 - 45]  *n* = 571 | 8.84 ± 7.81 | 7.44 ± 6.64 | 8.78 ± 7.23 | 12.5 ± 9.87 | 10.3 ± 9.26 | 7.78 ± 5.55 |
| [45 - 50]  *n* = 735 | 6.95 ± 6.52 | 8.93 ± 7.54 | 8.49 ± 6.96 | 12 ± 8.28 | 9.44 ± 7.62 | 7.14 ± 5.07 |
| [50 - 55]  *n* = 978 | 6.68 ± 5.29 | 8.4 ± 6.61 | 7.64 ± 6.51 | 11.6 ± 7.53 | 9.66 ± 6.81 | 5.99 ± 4.56 |
| [55 - 60]  *n* = 1,236 | 8.71 ± 6.2 | 9.23 ± 6.43 | 8.65 ± 6.91 | 11.4 ± 6.85 | 10 ± 6.37 | 6.22 ± 4.63 |
| [60 - 65]  *n* = 978 | 11.1 ± 7.19 | 9.25 ± 6.54 | 8.46 ± 6.38 | 10.3 ± 6.65 | 10.2 ± 6.68 | 5.93 ± 4.3 |
| [65 - 70]  *n* = 1,183 | 14.1 ± 7.97 | 9.26 ± 6.19 | 8.57 ± 6.11 | 10.2 ± 7.07 | 10 ± 6.58 | 6.15 ± 4.29 |
| [70 - 75]  *n* = 1,755 | 15.9 ± 8.75 | 8.63 ± 6.02 | 8.02 ± 5.68 | 9.87 ± 7.45 | 7.84 ± 5.79 | 5.8 ± 4.01 |
| [75 - 80]  *n* = 1,399 | 18.9 ± 9.44 | 6.51 ± 5.55 | 5.4 ± 4.87 | 8.06 ± 8.19 | 4.69 ± 6.09 | 4.35 ± 3.49 |
| [80 - 85]  *n* = 687 | 22.1 ± 10.6 | 4.35 ± 6.3 | 3.65 ± 4.8 | 6.39 ± 9.02 | 5.4 ± 7.19 | 4.03 ± 4.46 |
| [85 - 90]  *n* = 123 | 26.3 ± 10.6 | 3.78 ± 4.21 | 3.57 ± 4.24 | 6.83 ± 10.9 | 7.29 ± 3.08 | 5.34 ± 3.58 |
| Average  *n* = 10,509 | 14.6 ± 5.91 | 6.77 ± 2.2 | 6.81 ± 1.84 | 10.4 ± 2.23 | 8.09 ± 1.89 | 5.8 ± 1.16 |

The mean absolute error is reported as mean ± standard deviation. The average MAE across age ranges weighs each age range equally regardless of sample size. Basic sleep measures denote a linear regression model with the following predictive variables: sex, body mass index, arousal index, apnea-hypopnea index, total sleep time, wake after sleep onset, and percentage of N1, N2, N3, and REM sleep.

**Supplementary Table 3: Mean absolute error of the Home Positive Airway Pressure study stratified by 5-year age intervals.**

| Age Range [years] | Basic sleep measures | (a) Central EEG | (b) EEG+EOG+EMG | (c) ECG | (d) Respiratory | (e) Ensemble – Avg. |
| --- | --- | --- | --- | --- | --- | --- |
| [20 - 25]  *n* = 6 | 17.3 ± 4.23 | 6.3 ± 7 | 9.38 ± 10.8 | 26.7 ± 17 | 23.9 ± 8.7 | 16.4 ± 5.43 |
| [25 - 30]  *n* = 8 | 17.4 ± 9.58 | 4.79 ± 3.72 | 9.3 ± 7.74 | 17.9 ± 14.2 | 23.6 ± 9.5 | 13.3 ± 3.77 |
| [30 - 35]  *n* = 17 | 14.7 ± 8.89 | 4.72 ± 7.09 | 8.94 ± 10 | 24.1 ± 15 | 15.1 ± 5.46 | 12.3 ± 5.76 |
| [35 - 40]  *n* = 31 | 12.6 ± 5.48 | 6.23 ± 4.25 | 10.7 ± 9.38 | 11.7 ± 11.1 | 11.6 ± 7.63 | 8.21 ± 4.65 |
| [40 - 45]  *n* = 24 | 6.5 ± 5.51 | 8.57 ± 6.41 | 7.76 ± 7.99 | 16 ± 12.6 | 10.7 ± 7.23 | 7.15 ± 5.14 |
| [45 - 50]  *n* = 29 | 10.3 ± 10.3 | 7.79 ± 7.92 | 11.1 ± 8.22 | 13.1 ± 7.66 | 7.48 ± 4.82 | 5.91 ± 5.87 |
| [50 - 55]  *n* = 29 | 5.37 ± 5.03 | 9.24 ± 7.25 | 10.3 ± 8.48 | 13.6 ± 7.92 | 6.24 ± 3.97 | 4.95 ± 4.17 |
| [55 - 60]  *n* = 25 | 9.35 ± 6.37 | 12 ± 7.7 | 8.5 ± 6.99 | 11.1 ± 6.88 | 7.64 ± 5.76 | 5.65 ± 4.26 |
| [60 - 65]  *n* = 9 | 11.9 ± 8.36 | 11.3 ± 6.56 | 12.3 ± 6.12 | 14.7 ± 8.1 | 9.19 ± 5.7 | 7.19 ± 3.64 |
| [65 - 70]  *n* = 3 | 15.2 ± 9.6 | 10.1 ± 1.45 | 4.5 ± 2.18 | 4.66 ± 4.06 | 13.8 ± 4.56 | 5.38 ± 3.69 |
| [70 - 75]  *n* = 8 | 17 ± 9.74 | 7.42 ± 2.51 | 8.95 ± 3.75 | 8.6 ± 6.4 | 15.2 ± 5.91 | 5.11 ± 3.51 |
| [75 - 80]  *n* = 0 | - | - | - | - | - | - |
| [80 - 85]  *n* = 1 | 12.9 ± 0 | 3.39 ± 0 | 1.68 ± 0 | 4.75 ± 0 | 19.4 ± 0 | 6.46 ± 0 |
| [85 - 90]  *n* = 0 | - | - | - | - | - | - |
| Average  *n* = 190 | 12.5 ± 4.06 | 7.65 ± 2.7 | 8.62 ± 2.92 | 13.9 ± 6.74 | 13.7 ± 6.05 | 8.16 ± 3.75 |

The mean absolute error is reported as mean ± standard deviation. The average MAE across age ranges weighs each age range equally regardless of sample size. Basic sleep measures denote a linear regression model with the following predictive variables: sex, body mass index, arousal index, apnea-hypopnea index, total sleep time, wake after sleep onset, and percentage of N1, N2, N3, and REM sleep.

**Supplementary Table 4: Association between AEEs from all models and basic sleep metrics.**

|  | **σ** | **(a)**  **Central EEG** | **(b)**  **EEG+EOG+EMG** | **(c)**  **ECG** | **(d)**  **Respiratory** | **(e)**  **Ensemble – Avg.** |
| --- | --- | --- | --- | --- | --- | --- |
| **TST**  **(*n* = 12,595)** | 69.8 | -0.21  (p = 0.015) | **-0.84**  **(p = 2.9e-27)** | -0.19  (p = 0.061) | -0.037  (p = 0.63) | **-0.32**  **(p = 7.2e-10)** |
| **WASO**  **(*n* = 12,593)** | 57.7 | **0.67**  **(p = 1.2e-12)** | **0.87**  **(p = 1.9e-24)** | **0.45**  **(p = 5.2e-05)** | 0.14  (p = 0.097) | **0.53**  **(p = 6.3e-21)** |
| **SE**  **(*n* = 12,595)** | 0.126 | -0.16  (p = 0.066) | **-0.94**  **(p = 1.2e-32)** | **-0.42**  **(p = 5.7e-05)** | -0.11  (p = 0.16) | **-0.41**  **(p = 1e-14)** |
| **N1 %**  **(*n* = 12,594)** | 6.74 | **0.49**  **(p = 1.7e-07)** | **0.62**  **(p = 3.3e-13)** | 0.28  (p = 0.011) | 0.24  (p = 0.0047) | **0.41**  **(p = 5.5e-13)** |
| **N2 %**  **(*n* = 12,594)** | 14.1 | **-0.43**  **(p = 5.1e-07)** | **-0.28**  **(p = 0.00033)** | -0.1  (p = 0.33) | 0.23  (p = 0.0034) | -0.15  (p = 0.0052) |
| **N3 %**  **(*n* = 12,594)** | 11.2 | **0.59**  **(p = 3.7e-10)** | 0.17  (p = 0.043) | 0.023  (p = 0.84) | **-0.3**  **(p = 0.00044)** | 0.12  (p = 0.034) |
| **REM %**  **(*n* = 12,594)** | 8.06 | -0.19  (p = 0.022) | -0.12  (p = 0.13) | 0.031  (p = 0.75) | -0.2  (p = 0.0078) | -0.12  (p = 0.018) |
| **ArI**  **(*n* = 12,477)** | 11.3 | **0.74**  **(p = 1.1e-16)** | **0.52**  **(p = 1.5e-10)** | 0.11  (p = 0.3) | **0.78**  **(p = 8.7e-22)** | **0.53**  **(p = 2.5e-23)** |
| **AHI**  **(*n* = 12,556)** | 16.6 | **0.43**  **(p = 2e-06)** | 0.15  (p = 0.066) | 0.25  (p = 0.024) | **1.5**  **(p = 4.7e-76)** | **0.59**  **(p = 1e-26)** |
| **PLMI**  **(*n* = 6,889)** | 30.7 | -0.0094  (p = 0.94) | 0.14  (p = 0.15) | 0.49  (p = 0.0024) | -0.14  (p = 0.13) | 0.12  (p = 0.093) |
| **SaO2-80**  **(*n* = 9,871)** | 9.01 | 0.33  (p = 0.003) | 0.026  (p = 0.79) | -0.28  (p = 0.024) | **0.36**  **(p = 0.00029)** | 0.11  (p = 0.097) |

The associations were tested using linear regression with AEE as a response variable while controlling for age, sex, body mass index, and cohort. The *p*-values were calculated using a two-sided t-test. Regression coefficients describe the average change in AEE for an increase of one standard deviation (σ) and are marked in **bold** as significant for (p < 0.0009), corresponding to a significance level of 0.05 with Bonferroni correction. **Red** and **blue** indicates significant associations that are **positive** and **negative**, respectively. AEE: age estimate error; ArI: arousal index; AHI; apnea-hypopnea index; PLMI: periodic leg movement index; TST: total sleep time; WASO: wake after sleep onset; SE: sleep efficiency; SaO2-80: sleep time with blood oxygen saturation below 80 %.

**Supplementary Table 5: Association of AEE to morbidities and medication.**

|  | **(a)**  **Central EEG** | **(b)**  **EEG** | **(c)**  **ECG** | **(d)**  **Respiratory** | **(e)**  **Ensemble – Avg.** |
| --- | --- | --- | --- | --- | --- |
| **Sex: Male**  ***n* = (7,625/ 12,595)** | 0.59  (p = 0.0016) | -0.49  (p = 0.0045) | **-1.9**  **(p = 6.1e-18)** | **3.6**  **(p = 3.5e-96)** | **0.44**  **(p = 0.0001)** |
| **Body mass index**  ***n* = (12,595)** | -0.11  (p = 0.22) | **-0.27**  **(p = 0.00055)** | 0.34  (p = 0.0011) | **1.2**  **(p = 2e-54)** | **0.3**  **(p = 1.4e-08)** |
| **Hypertension**  ***n* = (4,488 / 11,104)** | **1.4**  **(p = 1.8e-13)** | **0.86**  **(p = 8.5e-07)** | **2.2**  **(p = 8.8e-24)** | 0.31  (p = 0.076) | **1.2**  **(p = 1.6e-25)** |
| **Congestive heart failure**  ***n* = (318 / 9,674)** | 1.8  (p = 0.0013) | **1.7**  **(p = 0.0005)** | **3.1**  **(p = 5.1e-07)** | 0.99  (p = 0.045) | **1.9**  **(p = 3.4e-09)** |
| **Heart attack**  ***n* = (899/ 9,669)** | 0.78  (p = 0.018) | 0.34  (p = 0.25) | **1.8**  **(p = 3.3e-06)** | -0.15  (p = 0.63) | **0.7**  **(p = 0.00046)** |
| **Chronic obstructive pulmonary disease**  ***n* = (210 / 8,483)** | 0.24  (p = 0.72) | 0.45  (p = 0.46) | 0.5  (p = 0.52) | 0.77  (p = 0.21) | 0.49  (p = 0.22) |
| **Stroke**  ***n* = (297 / 9,674)** | 0.049  (p = 0.93) | 0.27  (p = 0.57) | 0.06  (p = 0.92) | 0.21  (p = 0.67) | 0.15  (p = 0.65) |
| **Type 2 Diabetes**  ***n* = (944/ 10,153)** | **1.6**  **(p = 9e-07)** | **1.2**  **(p = 7.7e-05)** | -0.2  (p = 0.6) | 0.13  (p = 0.68) | **0.68**  **(p = 0.00058)** |
| **Benzodiazepines**  ***n* = (507 / 9,659)** | -0.92  (p = 0.035) | -0.13  (p = 0.74) | 0.21  (p = 0.67) | 0.82  (p = 0.035) | -0.0028  (p = 0.99) |
| **Antidepressants**  ***n* = (934/ 10,405)** | 0.39  (p = 0.24) | 0.78  (p = 0.0079) | 0.58  (p = 0.12) | **1.2**  **(p = 0.00012)** | **0.73**  **(p = 0.0002)** |

The associations were tested using linear regression with AEE as a response variable while controlling for age, sex, body mass index, and cohort. The *p*-values were calculated using a two-sided t-test. Regression coefficients are marked in **bold** as significant for (p < 0.001), corresponding to a significance level of 0.05 with Bonferroni correction. **Red** and **blue** indicates significant associations that are **positive** and **negative**, respectively. Regression coefficient for body mass index is for an increase of one standard deviation (σ = 6.44).

**Supplementary Table 6: All-cause mortality analysis of selected variables.**

| Predictor | *n*  *(positive / total)* | σ | HR (95 % CI) |
| --- | --- | --- | --- |
| Age [years] | 9386 | 11.69 | 3.93 (3.70 - 4.18) |
| Sex (male) | 5969 / 9386 | 1.00 | 1.42 (1.28 - 1.58) |
| BMI [kg/m^2^] | 9386 | 5.10 | 1.07 (1.03 - 1.12) |
| Alcohol [drinks/day] | 8772 | 4.64 | 1.04 (0.99 - 1.08) |
| Caffeine [serving/day] | 9312 | 2.46 | 1.08 (1.02 - 1.14) |
| Medication use |  |  |  |
| Antidepressants | 817 / 9386 | - | 1.46 (1.29 - 1.65) |
| Benzodiazepines | 486 / 9370 | - | 1.32 (1.14 - 1.53) |
| Sedatives | 57 / 909 | - | - |
| Race white | 8232 / 9385 | - | 1.03 (0.91 - 1.17) |
| Smoking |  |  |  |
| Current | 737 / 9350 | - | 1.77 (1.52 - 2.07) |
| Past | 4395 / 9350 | - | 1.17 (1.09 - 1.26) |
| Education [years] |  |  |  |
| [11 – 15] | 3787 / 8940 | - | 1.22 (1.13 - 1.32) |
| [16 – 20] | 3717 / 8940 | - | 0.92 (0.85 - 0.99) |
| > 20 | 969 / 8940 | - | 0.80 (0.72 - 0.89) |
| Objective sleep measures |  |  |  |
| Actigraphy MSSWOSI | 2739 | 73.42 | 0.95 (0.90 - 1.00) |
| Actigraphy WASO | 2739 | 43.43 | 1.19 (1.13 - 1.24) |
| WASO [min] | 9386 | 57.11 | 1.10 (1.06 - 1.14) |
| N2 % | 9385 | 12.37 | 1.07 (1.03 - 1.11) |
| REM % | 9385 | 6.96 | 0.85 (0.82 - 0.88) |
| ArI [h^-1^] | 9369 | 11.16 | 1.02 (0.99 - 1.06) |
| AHI [h^-1^] | 9348 | 15.97 | 1.05 (1.01 - 1.09) |
| SaO2-80 [min] | 9368 | 7.87 | 1.07 (1.04 - 1.11) |
| Hypertension | 4082 / 9385 | - | 1.39 (1.29 - 1.50) |
| Congestive heart failure | 309 / 9385 | - | 2.15 (1.88 - 2.46) |
| Heart attack | 846 / 8661 | - | 1.61 (1.46 - 1.77) |
| Chronic obstructive pulmonary disease | 205 / 8383 | - | 1.59 (1.33 - 1.89) |
| Stroke | 278 / 8661 | - | 1.74 (1.49 - 2.04) |
| Type 2 diabetes | 806 / 9150 | - | 1.67 (1.51 - 1.84) |
| Epworth Sleepiness Scale | 9173 | - | 0.99 (0.95 - 1.03) |
| MMSE | 2779 | - | 0.83 (0.79 - 0.86) |
| PASE | 2780 | - | 0.85 (0.80 - 0.90) |

HR was estimated with Cox proportional hazards models for an increase of one for binary variables and one standard deviation for all other variables. The models were adjusted for age, sex, BMI, and cohort. These analyses were performed in the Sleep Heart Healthy Study, the MrOS Sleep Study, and the Wisconsin Sleep Cohort. HR: hazard ratio; σ: standard deviation; BMI: body mass index; MSSWOSI: mean scored sleep while outside of sleep interval; WASO: wake after sleep onset; SaO2-80: sleep time with blood oxygen saturation below 80 %; MMSE: Teng Mini Mental State Examination; PASE: Physical Activity Scale for the Elderly Score.

**Supplementary Table 7: Summary of baseline demographics, lifestyle, and health characteristics of the Sleep Heart Health Study by age estimate error quartiles (Q1-Q4).**

|  |  | Q1  AEEc ≤ -3.9  (*n* = 1,424) | Q2  -3.9 < AEEc ≤ 0.2  (*n* = 1,424) | Q3  0.2 < AEEc ≤ 4.2  (*n* = 1,424) | Q4  4.2 < AEEc  (*n* = 1,424) | *p*-value |
| --- | --- | --- | --- | --- | --- | --- |
| Age | **μ ± σ** | 61.8 ± 11.0 | 63.4 ± 12.3 | 65.3 ± 11.2 | 62.5 ± 9.91 | **9.5e-17** |
| Sex | **n, (%)** | 710, (49.9 %) | 652, (45.8 %) | 661, (46.4 %) | 694, (48.7 %) | 0.098 |
| Body mass index | **μ ± σ** | 28.0 ± 4.94 | 27.7 ± 4.92 | 28.1 ± 5.15 | 28.9 ± 5.28 | **3.2e-09** |
| Alcohol [drinks/day] | **μ ± σ** | 2.89 ± 5.25 | 2.71 ± 5.24 | 2.94 ± 6.21 | 2.82 ± 6.23 | 0.024 |
| Caffeine [serving/day] | **μ ± σ** | 2.63 ± 2.67 | 2.67 ± 2.76 | 2.61 ± 2.59 | 2.67 ± 2.65 | 0.95 |
| Medication use |  |  |  |  |  |  |
| Antidepressants | **n, (%)** | 87, (6.11 %) | 96, (6.74 %) | 97, (6.81 %) | 129, (9.06 %) | 0.014 |
| Benzodiazepines | **n, (%)** | 72, (5.09 %) | 65, (4.58 %) | 86, (6.05 %) | 75, (5.27 %) | 0.36 |
| Race white | **n, (%)** | 1146, (80.5 %) | 1170, (82.2 %) | 1244, (87.4 %) | 1267, (89.0 %) | **1.1e-11** |
| Smoking |  |  |  |  |  |  |
| Current | **n, (%)** | 124, (8.79 %) | 132, (9.32 %) | 141, (9.94 %) | 150, (10.6 %) | 0.39 |
| Past | **n, (%)** | 636, (45.1 %) | 567, (40.0 %) | 625, (44.0 %) | 630, (44.6 %) | 0.026 |
| Education [years] |  |  |  |  |  |  |
| [11 – 15] | **n, (%)** | 639, (51.0 %) | 609, (47.5 %) | 750, (55.3 %) | 718, (52.9 %) | **0.00071** |
| [16 – 20] | **n, (%)** | 485, (38.7 %) | 502, (39.2 %) | 453, (33.4 %) | 445, (32.8 %) | **0.00019** |
| > 20 | **n, (%)** | 55, (4.39 %) | 53, (4.14 %) | 45, (3.32 %) | 65, (4.79 %) | 0.27 |
| Objective sleep measures |  |  |  |  |  |  |
| WASO [min] | **μ ± σ** | 54.8 ± 40.1 | 60.2 ± 43.2 | 62.1 ± 41.1 | 69.2 ± 50.2 | **4.4e-16** |
| NREM 2 % | **μ ± σ** | 58.5 ± 12.4 | 57.9 ± 12.9 | 58.2 ± 13.0 | 57.9 ± 13.4 | 0.15 |
| REM % | **μ ± σ** | 19.9 ± 6.98 | 19.9 ± 6.85 | 19.3 ± 6.92 | 19.2 ± 7.19 | 0.027 |
| ArI [h^-1^] | **μ ± σ** | 17.7 ± 9.37 | 19.0 ± 10.3 | 19.7 ± 11.1 | 20.3 ± 11.5 | **5.2e-09** |
| AHI [h^-1^] | **μ ± σ** | 15.3 ± 14.2 | 17.2 ± 15.5 | 19.0 ± 16.6 | 20.3 ± 17.7 | **7.1e-19** |
| SaO2-80 | **μ ± σ** | 0.245 ± 2.15 | 0.912 ± 11.4 | 0.557 ± 5.74 | 0.954 ± 8.30 | **4.2e-06** |
| Hypertension | **n, (%)** | 510, (35.8 %) | 562, (39.5 %) | 651, (45.7 %) | 707, (49.6 %) | **1.8e-14** |
| Congestive heart failure | **n, (%)** | 20, (1.41 %) | 32, (2.25 %) | 40, (2.81 %) | 45, (3.16 %) | **0.014** |
| Heart attack | **n, (%)** | 64, (5.36 %) | 74, (5.95 %) | 104, (8.23 %) | 94, (7.39 %) | **0.017** |
| COPD | **n, (%)** | 13, (0.928 %) | 13, (0.925 %) | 14, (0.999 %) | 20, (1.43 %) | 0.5 |
| Stroke | **n, (%)** | 26, (2.18 %) | 37, (2.98 %) | 41, (3.25 %) | 49, (3.85 %) | 0.11 |
| Type 2 diabetes | **n, (%)** | 92, (6.80 %) | 88, (6.49 %) | 102, (7.43 %) | 119, (8.64 %) | 0.14 |
| Epworth Sleepiness Scale | **μ ± σ** | 7.76 ± 4.38 | 7.68 ± 4.40 | 7.77 ± 4.39 | 7.83 ± 4.41 | 0.81 |

The AEE quartiles were from model (e, Ensemble – Avg.). Statistical comparisons were evaluated using chi-squared test for binary variables and Kruskal-Wallis tests for continuous distributions. WASO: wake after sleep onset; ArI: arousal index; AHI: apnea-hypopnea index; SaO2-80: sleep time with blood oxygen saturation below 80 %; COPD: Chronic obstructive pulmonary disease.

**Supplementary Table 8: Summary of baseline demographics, lifestyle, and health characteristics of the Wisconsin Sleep Cohort by age estimate error quartiles (Q1-Q4).**

|  |  | Q1  AEEc ≤ -2.7  (*n* = 229) | Q2  -2.7 < AEEc ≤ 0.3  (*n* = 228) | Q3  0.3 < AEEc ≤ 2.9  (*n* = 229) | Q4  2.9 < AEEc  (*n* = 229) | *p*-value |
| --- | --- | --- | --- | --- | --- | --- |
| Age | **μ ± σ** | 54.8 ± 7.20 | 54.9 ± 7.91 | 56.2 ± 7.32 | 55.8 ± 7.78 | 0.17 |
| Sex | **n, (%)** | 96, (42.1 %) | 114, (49.8 %) | 131, (57.5 %) | 132, (57.6 %) | **0.0022** |
| Body mass index | **μ ± σ** | 32.4 ± 7.04 | 31.3 ± 7.35 | 31.0 ± 6.40 | 31.0 ± 6.76 | 0.092 |
| Alcohol [drinks/day] | **μ ± σ** | 0.260 ± 0.596 | 0.301 ± 0.508 | 0.510 ± 0.867 | 0.340 ± 0.539 | **0.00057** |
| Caffeine [serving/day] | **μ ± σ** | 2.63 ± 2.24 | 2.62 ± 1.95 | 2.76 ± 2.27 | 2.72 ± 2.18 | 0.82 |
| Medication use |  |  |  |  |  |  |
| Antidepressants | **n, (%)** | 52, (22.8 %) | 56, (24.5 %) | 40, (17.5 %) | 41, (17.9 %) | 0.17 |
| Benzodiazepines | **n, (%)** | 17, (7.46 %) | 24, (10.5 %) | 13, (5.70 %) | 13, (5.68 %) | 0.16 |
| Sedatives | **n, (%)** | 15, (6.58 %) | 21, (9.17 %) | 12, (5.26 %) | 10, (4.37 %) | 0.17 |
| Race white | **n, (%)** | 212, (93.8 %) | 214, (94.7 %) | 225, (98.7 %) | 222, (97.4 %) | **0.023** |
| Smoking |  |  |  |  |  |  |
| Current | **n, (%)** | 25, (11.0 %) | 45, (19.9 %) | 31, (13.6 %) | 33, (14.5 %) | 0.056 |
| Past | **n, (%)** | 72, (31.7 %) | 76, (33.6 %) | 89, (39.0 %) | 79, (34.6 %) | 0.41 |
| Education [years] |  |  |  |  |  |  |
| [11 – 15] | **n, (%)** | 127, (55.9 %) | 134, (59.3 %) | 126, (55.3 %) | 117, (51.3 %) | 0.4 |
| [16 – 20] | **n, (%)** | 99, (43.6 %) | 91, (40.3 %) | 99, (43.4 %) | 110, (48.2 %) | 0.39 |
| > 20 | **n, (%)** | 1, (0.441 %) | 1, (0.442 %) | 3, (1.32 %) | 1, (0.439 %) | 0.57 |
| Objective sleep measures |  |  |  |  |  |  |
| WASO [min] | **μ ± σ** | 59.3 ± 37.2 | 66.1 ± 41.7 | 74.0 ± 49.3 | 80.1 ± 50.0 | **1.1e-05** |
| NREM 2 % | **μ ± σ** | 69.8 ± 8.03 | 70.1 ± 7.54 | 68.7 ± 8.78 | 68.8 ± 9.68 | 0.33 |
| REM % | **μ ± σ** | 17.1 ± 6.20 | 16.8 ± 6.21 | 16.8 ± 6.49 | 15.5 ± 6.45 | **0.021** |
| ArI [h^-1^] | **μ ± σ** | 20.5 ± 8.28 | 23.1 ± 9.81 | 23.1 ± 9.53 | 26.5 ± 13.3 | **1.7e-05** |
| AHI [h^-1^] | **μ ± σ** | 5.20 ± 8.53 | 6.72 ± 9.59 | 5.70 ± 7.18 | 9.99 ± 12.3 | **0.00031** |
| SaO2-80 | **μ ± σ** | 6.60 ± 14.0 | 6.22 ± 12.1 | 7.08 ± 13.4 | 8.58 ± 19.7 | 0.1 |
| Hypertension | **n, (%)** | 53, (23.2 %) | 62, (27.1 %) | 73, (32.0 %) | 84, (36.7 %) | **0.013** |
| Congestive heart failure | **n, (%)** | 0, (0.000 %) | 2, (0.873 %) | 2, (0.877 %) | 4, (1.75 %) | 0.26 |
| Heart attack | **n, (%)** | 3, (1.32 %) | 4, (1.75 %) | 7, (3.07 %) | 16, (6.99 %) | **0.0023** |
| Stroke | **n, (%)** | 4, (1.75 %) | 0, (0.000 %) | 6, (2.63 %) | 6, (2.62 %) | 0.11 |
| Type 2 diabetes | **n, (%)** | 2, (0.881 %) | 10, (4.42 %) | 12, (5.26 %) | 12, (5.26 %) | 0.05 |
| Epworth Sleepiness Scale | **μ ± σ** | 9.31 ± 4.42 | 9.10 ± 4.25 | 8.75 ± 4.11 | 8.96 ± 4.24 | 0.73 |

The AEE quartiles were from model (e, Ensemble – Avg.). Statistical comparisons were evaluated using chi-squared test for binary variables and Kruskal-Wallis tests for continuous distributions. WASO: wake after sleep onset; ArI: arousal index; AHI: apnea-hypopnea index; SaO2-80: sleep time with blood oxygen saturation below 80 %.

**Supplementary Table 9: Summary of baseline demographics, lifestyle, and health characteristics of the MrOS Sleep Study by age estimate error quartiles (Q1-Q4).**

|  |  | Q1  AEEc ≤ -1.9  (*n* = 713) | Q2  -1.9 < AEEc ≤ 1.3  (*n* = 714) | Q3  1.3 < AEEc ≤ 3.3  (*n* = 713) | Q4  3.3 < AEEc  (*n* = 714) | *p*-value |
| --- | --- | --- | --- | --- | --- | --- |
| Age | **μ ± σ** | 75.2 ± 5.14 | 78.1 ± 6.14 | 77.6 ± 5.09 | 74.0 ± 3.50 | **2e-54** |
| Body mass index | **μ ± σ** | 27.2 ± 3.87 | 27.0 ± 3.78 | 26.9 ± 3.63 | 27.6 ± 3.92 | **0.0055** |
| Alcohol [drinks/day] | **μ ± σ** | 0.481 ± 0.620 | 0.483 ± 0.614 | 0.531 ± 0.641 | 0.499 ± 0.608 | 0.51 |
| Caffeine [serving/day] | **μ ± σ** | 0.246 ± 0.647 | 0.220 ± 0.554 | 0.223 ± 0.585 | 0.230 ± 0.564 | 0.93 |
| Medication use |  |  |  |  |  |  |
| Antidepressants | **n, (%)** | 56, (7.85 %) | 55, (7.71 %) | 54, (7.56 %) | 58, (8.13 %) | 0.98 |
| Benzodiazepines | **n, (%)** | 39, (5.47 %) | 36, (5.05 %) | 24, (3.36 %) | 29, (4.07 %) | 0.21 |
| Race white | **n, (%)** | 629, (88.2 %) | 655, (91.9 %) | 658, (92.2 %) | 655, (91.9 %) | **0.027** |
| Smoking |  |  |  |  |  |  |
| Current | **n, (%)** | 17, (2.38 %) | 13, (1.82 %) | 13, (1.82 %) | 14, (1.96 %) | 0.86 |
| Past | **n, (%)** | 412, (57.8 %) | 404, (56.7 %) | 406, (56.9 %) | 439, (61.6 %) | 0.21 |
| Education [years] |  |  |  |  |  |  |
| [11 – 15] | **n, (%)** | 137, (19.2 %) | 162, (22.7 %) | 148, (20.7 %) | 126, (17.7 %) | 0.1 |
| [16 – 20] | **n, (%)** | 370, (51.9 %) | 364, (51.1 %) | 357, (50.0 %) | 381, (53.4 %) | 0.61 |
| > 20 | **n, (%)** | 195, (27.3 %) | 178, (25.0 %) | 194, (27.2 %) | 202, (28.3 %) | 0.54 |
| Objective sleep measures |  |  |  |  |  |  |
| Actigraphy MSSWOSI | μ ± σ | 384.2 ± 73.3 | 382.0 ± 75.1 | 385.7 ± 75.0 | 389.6 ± 69.3 | 0.28 |
| Actigraphy WASO | μ ± σ | 74.3 ± 41.6 | 79.6 ± 45.8 | 77.9 ± 42.8 | 79.5 ± 43.3 | 0.067 |
| WASO [min] | **μ ± σ** | 111.3 ± 69.4 | 114.3 ± 63.1 | 117.9 ± 67.3 | 116.3 ± 65.3 | 0.09 |
| NREM 2 % | **μ ± σ** | 64.0 ± 11.0 | 63.2 ± 9.65 | 62.6 ± 10.4 | 62.6 ± 9.73 | 0.077 |
| REM % | **μ ± σ** | 19.2 ± 7.33 | 19.0 ± 6.67 | 19.0 ± 6.94 | 19.5 ± 6.60 | 0.46 |
| ArI [h^-1^] | **μ ± σ** | 22.8 ± 11.4 | 24.0 ± 12.1 | 23.8 ± 11.4 | 23.6 ± 11.7 | 0.11 |
| AHI [h^-1^] | **μ ± σ** | 19.6 ± 15.7 | 20.8 ± 16.1 | 20.6 ± 15.3 | 21.3 ± 15.9 | 0.17 |
| SaO2-80 | **μ ± σ** | 0.374 ± 2.17 | 0.349 ± 2.07 | 0.263 ± 1.56 | 0.269 ± 1.71 | 0.5 |
| Hypertension | **n, (%)** | 302, (42.4 %) | 343, (48.1 %) | 366, (51.3 %) | 405, (56.8 %) | **8.8e-07** |
| Congestive heart failure | **n, (%)** | 20, (2.81 %) | 41, (5.75 %) | 33, (4.63 %) | 72, (10.1 %) | **3.5e-08** |
| Heart attack | **n, (%)** | 83, (11.6 %) | 137, (19.2 %) | 125, (17.5 %) | 151, (21.2 %) | **1.3e-05** |
| COPD | **n, (%)** | 34, (4.77 %) | 36, (5.05 %) | 42, (5.89 %) | 37, (5.19 %) | 0.8 |
| Stroke | **n, (%)** | 33, (4.63 %) | 27, (3.79 %) | 29, (4.07 %) | 21, (2.95 %) | 0.42 |
| Type 2 diabetes | **n, (%)** | 88, (12.3 %) | 87, (12.2 %) | 89, (12.5 %) | 111, (15.6 %) | 0.19 |
| Epworth Sleepiness Scale | **μ ± σ** | 6.29 ± 3.89 | 6.01 ± 3.44 | 6.03 ± 3.64 | 6.23 ± 3.66 | 0.67 |
| MMSE | **μ ± σ** | 92.9 ± 6.02 | 92.7 ± 6.14 | 92.1 ± 6.00 | 93.5 ± 5.17 | **3.6e-05** |
| PASE | **μ ± σ** | 151.5 ± 73.3 | 143.0 ± 70.3 | 144.5 ± 70.4 | 146.8 ± 70.8 | 0.099 |

The AEE quartiles were from model (e, Ensemble – Avg.). Statistical comparisons were evaluated using chi-squared test for binary variables and Kruskal-Wallis tests for continuous distributions. MSSWOSI: mean scored sleep while outside of sleep interval; WASO: wake after sleep onset; ArI: arousal index; AHI: apnea-hypopnea index; SaO2-80: sleep time with blood oxygen saturation below 80 %; MMSE: Teng Mini Mental State Examination; PASE: Physical Activity Scale for the Elderly Score.

**Supplementary Table 10: Mortality hazard ratios per 10-year increment in AEE in the Sleep Heart Health Study.**

|  |  | Cox Model 1  HR (95% CI) | Cox Model 2  HR (95% CI) | Cox Model 3  HR (95% CI) |
| --- | --- | --- | --- | --- |
| All-cause | (a) Central EEG | 1.11 (1.04 - 1.17) | 1.11 (1.05 - 1.18) | 1.06 (1.00 - 1.13) |
|  | (b) EEG+EOG+EMG | 1.18 (1.10 - 1.26) | 1.18 (1.11 - 1.26) | 1.14 (1.07 - 1.22) |
|  | (c) ECG | 1.06 (1.00 - 1.12) | 1.08 (1.02 - 1.15) | 1.04 (0.98 - 1.10) |
|  | (d) Respiratory | 1.16 (1.10 - 1.24) | 1.14 (1.07 - 1.21) | 1.11 (1.04 - 1.19) |
|  | (e) Ensemble – Avg. | 1.39 (1.25 - 1.54) | 1.39 (1.25 - 1.54) | 1.26 (1.13 - 1.40) |
| Cardiovascular | (a) Central EEG | 1.19 (1.05 - 1.36) | 1.20 (1.05 - 1.36) | 1.13 (1.00 - 1.29) |
|  | (b) EEG+EOG+EMG | 1.21 (1.06 - 1.39) | 1.22 (1.06 - 1.39) | 1.16 (1.01 - 1.33) |
|  | (c) ECG | 1.25 (1.10 - 1.43) | 1.26 (1.11 - 1.44) | 1.18 (1.04 - 1.34) |
|  | (d) Respiratory | 1.16 (1.02 - 1.31) | 1.12 (0.99 - 1.27) | 1.09 (0.96 - 1.24) |
|  | (e) Ensemble – Avg. | 1.66 (1.34 - 2.06) | 1.64 (1.32 - 2.03) | 1.44 (1.16 - 1.79) |

The mortality analysis was performed with (*n* = 5,696, deaths = 1,285) for all-cause mortality and (*n* = 5,572, death = 357) for cardiovascular mortality. HR: hazard ratio. AEE: age estimate error. Model 1: age. Model 2: age, sex, body mass index, race, smoking status, education level, daily alcohol intake, daily caffeine intake, benzodiazepines, and antidepressants. Model 3: Model 1 + wake after sleep onset, N2 %, REM %, arousal index, apnea-hypopnea index, sleep time with blood oxygen saturation below 80 %, Epworth Sleepiness Scale Score, hypertension, congestive heart failure, history of heart attack, stroke, chronic obstructive pulmonary disease, and type 2 diabetes.

**Supplementary Table 11: Mortality hazard ratios per 10-year increment in AEE in the Wisconsin Sleep Cohort.**

|  |  | Cox Model 1  HR (95% CI) | Cox Model 2  HR (95% CI) | Cox Model 3  HR (95% CI) |
| --- | --- | --- | --- | --- |
| All-cause | (a) Central EEG | 1.52 (1.15 - 2.02) | 1.51 (1.14 - 2.00) | 1.24 (0.93 - 1.66) |
|  | (b) EEG+EOG+EMG | 1.00 (0.70 - 1.41) | 1.15 (0.80 - 1.65) | 0.85 (0.57 - 1.26) |
|  | (c) ECG | 0.71 (0.54 - 0.92) | 0.71 (0.55 - 0.93) | 0.66 (0.50 - 0.86) |
|  | (d) Respiratory | 0.95 (0.69 - 1.32) | 0.76 (0.53 - 1.08) | 0.61 (0.42 - 0.90) |
|  | (e) Ensemble – Avg. | 1.02 (0.60 - 1.72) | 0.98 (0.57 - 1.67) | 0.54 (0.30 - 0.98) |
| Cardiovascular | (a) Central EEG | 2.08 (1.15 - 3.78) | 2.19 (1.20 - 3.99) | 1.10 (0.58 - 2.08) |
|  | (b) EEG+EOG+EMG | 0.92 (0.45 - 1.86) | 1.39 (0.65 - 2.97) | 0.74 (0.32 - 1.69) |
|  | (c) ECG | 0.68 (0.40 - 1.17) | 0.68 (0.39 - 1.19) | 0.70 (0.38 - 1.28) |
|  | (d) Respiratory | 0.87 (0.45 - 1.66) | 0.55 (0.28 - 1.06) | 0.45 (0.21 - 0.97) |
|  | (e) Ensemble – Avg. | 1.16 (0.60 - 2.24) | 1.22 (0.61 - 2.45) | 0.88 (0.40 - 1.93) |

The mortality analysis was performed with (*n* = 909, deaths = 98) for all-cause mortality and (*n* = 835, death = 24) for cardiovascular mortality. HR: hazard ratio. AEE: age estimate error. Model 1: age. Model 2: age, sex, body mass index, race, smoking status, education level, daily alcohol intake, daily caffeine intake, benzodiazepines, sedatives, and antidepressants. Model 3: Model 1 + wake after sleep onset, N2 %, REM %, arousal index, apnea-hypopnea index, sleep time with blood oxygen saturation below 80 %, Epworth Sleepiness Scale Score, hypertension, congestive heart failure, history of heart attack, stroke, and type 2 diabetes.

**Supplementary Table 12: Mortality hazard ratios per 10-year increment in AEE in the MrOS Sleep Study.**

|  |  | Cox Model 1  HR (95% CI) | Cox Model 2  HR (95% CI) | Cox Model 3  HR (95% CI) |
| --- | --- | --- | --- | --- |
| All-cause | (a) Central EEG | 1.17 (1.09 - 1.26) | 1.19 (1.11 - 1.28) | 1.15 (1.07 - 1.24) |
|  | (b) EEG+EOG+EMG | 1.15 (1.04 - 1.28) | 1.18 (1.06 - 1.31) | 1.13 (1.01 - 1.25) |
|  | (c) ECG | 1.12 (1.07 - 1.17) | 1.12 (1.07 - 1.17) | 1.10 (1.05 - 1.15) |
|  | (d) Respiratory | 1.02 (0.85 - 1.23) | 1.03 (0.86 - 1.24) | 1.01 (0.85 - 1.21) |
|  | (e) Ensemble – Avg. | 1.45 (1.28 - 1.63) | 1.46 (1.29 - 1.65) | 1.37 (1.21 - 1.54) |
| Cardiovascular | (a) Central EEG | 1.23 (1.08 - 1.40) | 1.24 (1.09 - 1.41) | 1.16 (1.02 - 1.32) |
|  | (b) EEG+EOG+EMG | 1.12 (0.94 - 1.33) | 1.17 (0.98 - 1.39) | 1.09 (0.91 - 1.30) |
|  | (c) ECG | 1.15 (1.07 - 1.25) | 1.14 (1.05 - 1.23) | 1.09 (1.01 - 1.18) |
|  | (d) Respiratory | 0.85 (0.67 - 1.07) | 0.84 (0.66 - 1.07) | 0.80 (0.64 - 1.02) |
|  | (e) Ensemble – Avg. | 1.51 (1.22 - 1.86) | 1.51 (1.22 - 1.86) | 1.30 (1.05 - 1.60) |

The mortality analysis was performed with (*n* = 2,781, deaths = 1,662) for all-cause mortality and (*n* = 2,781, death = 595) for cardiovascular mortality. HR: hazard ratio. AEE: age estimate error. Model 1: age. Model 2: age, sex, body mass index, race, smoking status, education level, daily alcohol intake, daily caffeine intake, benzodiazepines, and antidepressants. Model 3: Model 1 + actigraohy wake after sleep onset, actigraphy mean scored sleep while outside of sleep interval, wake after sleep onset, N2 %, REM %, arousal index, apnea-hypopnea index, sleep time with blood oxygen saturation below 80 %, Epworth Sleepiness Scale Score, hypertension, congestive heart failure, history of heart attack, stroke, chronic obstructive pulmonary disease, and type 2 diabetes.

**Supplementary Table 13: Sensitivity analysis using only subjects without hypertension of mortality hazard ratios per 10-year increment in AEE in the combined data of the Sleep Heart Health Study, the Wisconsin Sleep Cohort, and the MrOS Sleep Study.**

|  |  | Cox Model 1  HR (95% CI) | Cox Model 2  HR (95% CI) | Cox Model 3  HR (95% CI) |
| --- | --- | --- | --- | --- |
| All-cause | (a) Central EEG | 1.13 (1.05 - 1.21) | 1.16 (1.08 - 1.24) | 1.12 (1.05 - 1.20) |
|  | (b) EEG+EOG+EMG | 1.07 (0.99 - 1.15) | 1.14 (1.05 - 1.24) | 1.12 (1.03 - 1.22) |
|  | (c) ECG | 1.04 (0.99 - 1.09) | 1.05 (1.00 - 1.11) | 1.04 (0.99 - 1.10) |
|  | (d) Respiratory | 1.01 (0.95 - 1.08) | 1.04 (0.96 - 1.14) | 1.07 (0.98 - 1.17) |
|  | (e) Ensemble – Avg. | 1.15 (1.04 - 1.27) | 1.28 (1.15 - 1.44) | 1.25 (1.11 - 1.40) |
| Cardiovascular | (a) Central EEG | 1.21 (1.04 - 1.40) | 1.28 (1.09 - 1.49) | 1.21 (1.03 - 1.41) |
|  | (b) EEG+EOG+EMG | 0.97 (0.84 - 1.13) | 1.07 (0.90 - 1.26) | 1.04 (0.88 - 1.23) |
|  | (c) ECG | 1.11 (1.00 - 1.22) | 1.12 (1.01 - 1.24) | 1.09 (0.98 - 1.21) |
|  | (d) Respiratory | 0.93 (0.81 - 1.06) | 0.96 (0.80 - 1.15) | 1.01 (0.84 - 1.21) |
|  | (e) Ensemble – Avg. | 1.17 (0.95 - 1.43) | 1.39 (1.09 - 1.76) | 1.31 (1.03 - 1.66) |

The mortality analysis was performed with (*n* = 5,303, deaths = 1,291) for all-cause mortality and (*n* = 5,224, death = 348) for cardiovascular mortality. HR: hazard ratio. AEE: age estimate error. Model 1: age. Model 2: age, sex, body mass index, race, smoking status, education level, daily alcohol intake, daily caffeine intake, benzodiazepines, sedatives, antidepressants, and cohort. Model 3: Model 2 + wake after sleep onset, N2 %, REM %, arousal index, apnea-hypopnea index, sleep time with blood oxygen saturation below 80 %, Epworth Sleepiness Scale Score, congestive heart failure, history of heart attack, stroke, and type 2 diabetes.

**Supplementary Table 14: Sensitivity analysis using only subjects without sleep apnea (AHI ≥ 15) of mortality hazard ratios per 10-year increment in AEE in the combined data of the Sleep Heart Health Study, the Wisconsin Sleep Cohort, and the MrOS Sleep Study.**

|  |  | Cox Model 1  HR (95% CI) | Cox Model 2  HR (95% CI) | Cox Model 3  HR (95% CI) |
| --- | --- | --- | --- | --- |
| All-cause | (a) Central EEG | 1.12 (1.05 - 1.20) | 1.14 (1.07 - 1.22) | 1.09 (1.02 - 1.16) |
|  | (b) EEG+EOG+EMG | 1.06 (0.99 - 1.14) | 1.10 (1.01 - 1.18) | 1.08 (1.00 - 1.16) |
|  | (c) ECG | 1.07 (1.02 - 1.12) | 1.07 (1.02 - 1.13) | 1.05 (1.00 - 1.10) |
|  | (d) Respiratory | 1.06 (1.00 - 1.12) | 1.09 (1.01 - 1.18) | 1.09 (1.01 - 1.18) |
|  | (e) Ensemble – Avg. | 1.20 (1.10 - 1.32) | 1.31 (1.17 - 1.46) | 1.22 (1.10 - 1.37) |
| Cardiovascular | (a) Central EEG | 1.22 (1.07 - 1.39) | 1.25 (1.09 - 1.43) | 1.16 (1.01 - 1.32) |
|  | (b) EEG+EOG+EMG | 1.03 (0.90 - 1.17) | 1.06 (0.92 - 1.23) | 1.03 (0.89 - 1.19) |
|  | (c) ECG | 1.14 (1.03 - 1.25) | 1.15 (1.04 - 1.27) | 1.09 (0.99 - 1.20) |
|  | (d) Respiratory | 1.01 (0.91 - 1.13) | 1.02 (0.88 - 1.18) | 1.01 (0.87 - 1.16) |
|  | (e) Ensemble – Avg. | 1.27 (1.06 - 1.51) | 1.43 (1.15 - 1.76) | 1.24 (1.01 - 1.54) |

The mortality analysis was performed with (*n* = 5,161, deaths = 1,390) for all-cause mortality and (*n* = 5,045, death = 424) for cardiovascular mortality. HR: hazard ratio. AEE: age estimate error. Model 1: age. Model 2: age, sex, body mass index, race, smoking status, education level, daily alcohol intake, daily caffeine intake, benzodiazepines, sedatives, antidepressants, and cohort. Model 3: Model 2 + wake after sleep onset, N2 %, REM %, arousal index, apnea-hypopnea index, sleep time with blood oxygen saturation below 80 %, Epworth Sleepiness Scale Score, hypertension, congestive heart failure, history of heart attack, stroke, and type 2 diabetes.

**Supplementary Table 15: Mortality hazard ratios per 10-year increment in AEE in the age estimation test set of the combined data of the Sleep Heart Health Study, the Wisconsin Sleep Cohort, and the MrOS Sleep Study.**

|  |  | Cox Model 1  HR (95% CI) | Cox Model 2  HR (95% CI) | Cox Model 3  HR (95% CI) |
| --- | --- | --- | --- | --- |
| All-cause | (a) Central EEG | 1.11 (1.06 - 1.17) | 1.14 (1.09 - 1.19) | 1.09 (1.04 - 1.14) |
|  | (b) EEG+EOG+EMG | 1.11 (1.05 - 1.16) | 1.17 (1.10 - 1.23) | 1.13 (1.07 - 1.20) |
|  | (c) ECG | 1.08 (1.05 - 1.12) | 1.10 (1.06 - 1.14) | 1.07 (1.03 - 1.11) |
|  | (d) Respiratory | 1.04 (0.99 - 1.09) | 1.09 (1.02 - 1.15) | 1.08 (1.01 - 1.14) |
|  | (e) Ensemble – Avg. | 1.23 (1.15 - 1.32) | 1.37 (1.26 - 1.48) | 1.27 (1.18 - 1.38) |
| Cardiovascular | (a) Central EEG | 1.17 (1.07 - 1.28) | 1.21 (1.11 - 1.33) | 1.14 (1.04 - 1.24) |
|  | (b) EEG+EOG+EMG | 1.10 (0.99 - 1.21) | 1.18 (1.06 - 1.31) | 1.13 (1.01 - 1.25) |
|  | (c) ECG | 1.14 (1.07 - 1.22) | 1.16 (1.08 - 1.24) | 1.10 (1.03 - 1.18) |
|  | (d) Respiratory | 1.03 (0.94 - 1.12) | 1.08 (0.96 - 1.22) | 1.07 (0.95 - 1.20) |
|  | (e) Ensemble – Avg. | 1.32 (1.16 - 1.50) | 1.53 (1.32 - 1.78) | 1.35 (1.16 - 1.56) |

The mortality analysis was performed with (*n* = 8,432, deaths = 2,601) for all-cause mortality and (*n* = 8,255, death = 823) for cardiovascular mortality. HR: hazard ratio. AEE: age estimate error. Model 1: age. Model 2: age, sex, body mass index, race, smoking status, education level, daily alcohol intake, daily caffeine intake, benzodiazepines, sedatives, antidepressants, and cohort. Model 3: Model 2 + wake after sleep onset, N2 %, REM %, arousal index, apnea-hypopnea index, sleep time with blood oxygen saturation below 80 %, Epworth Sleepiness Scale Score, hypertension, congestive heart failure, history of heart attack, stroke, and type 2 diabetes.

**Supplementary Table 16: Pseudo code for algorithm for uniform age sampling.**

| **Uniform age sampling strategy** |
| --- |
| Let *X* be the complete dataset.  Define *N* as the number of age bins.  Define *M* as the desired number of samples in the training set.  Let *X_train_* = *X* be the training subset. |
| **while** len(*X_train_*) > M do  Let *X_a_* be samples in *X_train_* in the age bin with most samples (if multiple choose one randomly).  Let *X_b_* be samples in *X_a_* that matches the cohort with most samples (if multiple choose one randomly).  Let *X_c_* be samples in *X_b_* with the majority sex (if equal choose one randomly).  Let *x_remove_* be a random sample in *X_c_*.  *X_train_* = *X_train_\ X_remove_* (*X_remove_* is removed from the *X_train_* set).  **end** |

The pseudo code samples *M* from *X* such that the samples are uniform in age while being evenly split between sex and cohorts in each age bin.

**Supplementary Table 17: Summary metrics of polysomnography data used for training and testing the age estimation models.**

|  |  | CFS | STAGES | WSC | MrOS | SSC | SHHS | HomePAP |
| --- | --- | --- | --- | --- | --- | --- | --- | --- |
| N | n | 730 | 1536 | 1603 | 2,874 | 700 | 5,703 | 190 |
| N (train) | n, (%) | 521, (71.4 %) | 555, (36.0 %) | 267, (16.7 %) | 287, (9.986 %) | 423, (60.4 %) | 447, (7.8 %) | 0, (0 %) |
| N (val) | n, (%) | 17, (2.3 %) | 58, (3.8 %) | 26, (1.6 %) | 30, (1.044 %) | 28, (4.00 %) | 41, (0.72 %) | 0, (0 %) |
| N (test) | n, (%) | 192, (26.3 %) | 881, (57.5 %) | 805, (50.2 %) | 2,557, (89.0 %) | 249, (35.6 %) | 5,215, (91.4 %) | 190, (100 %) |
| N (test V2) | n, (%) | - | 42, (2.7 %) | 505, (31.503 %) | - | - | - | - |
| Age [years] | μ ± σ | 41.4 ± 19.4 | 45.9 ± 14.4 | 57.7 ± 8.0 | 76.2 ± 5.3 | 45.7 ± 14.4 | 63.23 ± 11.21 | 46.11 ± 12.0 |
| Sex (male) | n, (%) | 329, (45.1 %) | 724, (47.3 %) | 846, (52.8 %) | 2874, (100.000 %) | 408, (58.3 %) | 2723, (47.7 %) | 99, (52.105 %) |
| BMI [kg/m^2^] | μ ± σ | 32.4 ± 9.5 | 31.3 ± 8.7 | 31.8 ± 7.3 | 27.2 ± 3.8 | 27.09 ± 6.77 | 28.17 ± 5.09 | 36.93 ± 9.04 |
| TST [min] | μ ± σ | 373.1 ± 75.0 | 352.3 ± 80.1 | 364.8 ± 64.1 | 355.9 ± 69.1 | 363.7 ± 79.1 | 356.2 ± 64.7 | 335.5 ± 75.3 |
| WASO [min] | μ ± σ | 82.2 ± 65.8 | 61.7 ± 50.8 | 70.6 ± 45.0 | 115.1 ± 66.6 | 73.9 ± 53.8 | 61.5 ± 44.1 | 77.6 ± 56.0 |
| SE | μ ± σ | 0.787 ± 0.130 | 0.809 ± 0.139 | 0.800 ± 0.116 | 0.761 ± 0.120 | 0.780 ± 0.135 | 0.780 ± 0.122 | 0.782 ± 0.149 |
| NREM 1 % | μ ± σ | 5.18 ± 4.8 | 11.9 ± 10.60 | 10.3 ± 6.59 | 6.91 ± 4.7 | 11.91 ± 10.4 | 5.38 ± 4.02 | 15.72 ± 12.5 |
| NREM 2 % | μ ± σ | 56.8 ± 13.6 | 57.9 ± 16.9 | 66.7 ± 9.4 | 63.2 ± 10.2 | 63.2 ± 25.1 | 58.14 ± 13.0 | 52.7 ± 13.3 |
| NREM 3 % | μ ± σ | 20.1 ± 13.5 | 11.06 ± 10.6 | 6.49 ± 7.2 | 10.8 ± 8.6 | 9.0 ± 9.4 | 16.92 ± 11.27 | 13.6 ± 9.68 |
| REM % | μ ± σ | 18.0 ± 7.5 | 19.1 ± 13.2 | 16.5 ± 6.4 | 19.1 ± 6.9 | 16.55 ± 7.9 | 19.56 ± 6.99 | 18.05 ± 8.21 |
| ArI [h^-1^] | μ ± σ | 15.6 ± 10.0 | 21.3 ± 12.2 | 23.1 ± 10.3 | 23.6 ± 11.7 | 19.1 ± 8.8 | 19.2 ± 10.6 | 23.8 ± 18.1 |
| AHI [h^-1^] | μ ± σ | 13.2 ± 20.5 | 14.33 ± 17.1 | 7.1 ± 9.6 | 20.7 ± 15.9 | 13.7 ± 19.3 | 18.00 ± 16.1 | 14.0 ± 18.5 |
| PLMI [h^-1^] | μ ± σ | 8.05 ± 17.963 | - | 7.80 ± 16.9 | 35.5 ± 37.4 | 7.0 ± 17.670 | - | - |
| SaO2-80 [min] | μ ± σ | 2.93 ± 12.7 | - | 6.3 ± 12.489 | 0.32 ± 1.9 | - | 0.7 ± 7.74 | - |

Annotations of arousals was not available for STAGES, WSC, and SSC. Annotations of PLMs were not available for STAGES, SHHS, and HPAP. N: number of participants; V2: visit 2; BMI: body mass index; TST: total sleep time; SL: sleep latency; WASO: wake after sleep onset; SE: sleep efficiency; ArI: arousal index; AHI: apnea-hypopnea index; PLMI: periodic leg movement index; SaO2-80: sleep time with blood oxygen saturation below 80 %; CFS: Cleveland Family Study; STAGES: Stanford Technology Analytics and Genomics of Sleep; WSC: Wisconsin Sleep Cohort; MrOS: MrOS Sleep Study; SSC: Stanford Sleep Cohort; SHHS; Sleep Heart Health Study; HomePAP: Home Positive Airway Pressure Study.

**Supplementary References**

1. Ioffe S, Szegedy C. Batch Normalization: Accelerating Deep Network Training by Reducing Internal Covariate Shift. In: *Proceedings of the 32nd International Conference on Machine Learning*. PMLR; 2015:448-456.

2. Olesen AN, Jørgen Jennum P, Mignot E, Sorensen HBD. Automatic sleep stage classification with deep residual networks in a mixed-cohort setting. *Sleep*. 2021;44(1). doi:10.1093/sleep/zsaa161

3. Olesen AN, Chambon S, Thorey V, Jennum P, Mignot E, Sorensen HBD. Towards a Flexible Deep Learning Method for Automatic Detection of Clinically Relevant Multi-Modal Events in the Polysomnogram. In: *2019 41st Annual International Conference of the IEEE Engineering in Medicine and Biology Society (EMBC)*. IEEE; 2019:556-561. doi:10.1109/EMBC.2019.8856570

4. Chambon S, Thorey V, Arnal PJ, Mignot E, Gramfort A. DOSED: A deep learning approach to detect multiple sleep micro-events in EEG signal. *J Neurosci Methods*. 2019;321:64-78. doi:10.1016/j.jneumeth.2019.03.017

5. Sandler M, Howard A, Zhu M, Zhmoginov A, Chen L-C. MobileNetV2: Inverted Residuals and Linear Bottlenecks. In: *Proceedings of the IEEE Conference on Computer Vision and Pattern Recognition (CVPR)*. ; 2018:4510-4520.

6. Bianco S, Cadene R, Celona L, Napoletano P. Benchmark Analysis of Representative Deep Neural Network Architectures. *IEEE Access*. 2018;6:64270-64277. doi:10.1109/ACCESS.2018.2877890

7. Terzano MG, Parrino L, Sherieri A, et al. Atlas, rules, and recording techniques for the scoring of cyclic alternating pattern (CAP) in human sleep. *Sleep Med*. 2001;2:537-553. doi:10.1016/S1389-9457(01)00149-6

8. Yang Z, Yang D, Dyer C, He X, Smola A, Hovy E. Hierarchical Attention Networks for Document Classification. In: *Proceedings of the 2016 Conference of the North American Chapter of the Association for Computational Linguistics: Human Language Technologies*. Association for Computational Linguistics; 2016:1480-1489. doi:10.18653/v1/N16-1174

9. Bahdanau D, Cho KH, Bengio Y. Neural machine translation by jointly learning to align and translate. In: *3rd International Conference on Learning Representations, ICLR 2015 - Conference Track Proceedings*. International Conference on Learning Representations, ICLR; 2015.

10. Srivastava N, Hinton G, Krizhevsky A, Salakhutdinov R. Dropout: A Simple Way to Prevent Neural Networks from Overfitting. *J Mach Learn Res*. 2014;15:1929-1958.

11. LeCun Y, Bengio Y, Hinton G. Deep learning. *Nature*. 2015;521:436-444. doi:10.1038/nature14539

12. Shahriari B, Swersky K, Wang Z, Adams RP, de Freitas N. Taking the Human Out of the Loop: A Review of Bayesian Optimization. *Proc IEEE*. 2016;104:148-175. doi:10.1109/JPROC.2015.2494218
