# Supplementary material for: Age estimation from sleep studies using deep learning predicts life expectancy
Source: NPJ Digit Med. 2022 Jul 22;5:103. doi: 10.1038/s41746-022-00630-9 (PMC9307657; doi:10.1038/s41746-022-00630-9)
Supplement: Supplementary file 2 — Nature Reporting Summary [file 41746_2022_630_MOESM2_ESM.pdf]

nature research

Corresponding author(s): Andreas Brink-Kjaer, abk26@stanford.edu
Emmanuel Mignot, mignot@stanford.edu

Last updated by author(s): Aug 15, 2021

Reporting Summary

Nature Research wishes to improve the reproducibility of the work that we publish. This form provides structure for consistency and transparency in reporting. For further information on Nature Research policies, see our Editorial Policies and the Editorial Policy Checklist.

Statistics

For all statistical analyses, confirm that the following items are present in the figure legend, table legend, main text, or Methods section.

n/a	Confirmed	
☐	☒	The exact sample size (*n*) for each experimental group/condition, given as a discrete number and unit of measurement
☐	☒	A statement on whether measurements were taken from distinct samples or whether the same sample was measured repeatedly
☐	☒	The statistical test(s) used AND whether they are one- or two-sided<br>*Only common tests should be described solely by name; describe more complex techniques in the Methods section.*
☐	☒	A description of all covariates tested
☐	☒	A description of any assumptions or corrections, such as tests of normality and adjustment for multiple comparisons
☐	☒	A full description of the statistical parameters including central tendency (e.g. means) or other basic estimates (e.g. regression coefficient) AND variation (e.g. standard deviation) or associated estimates of uncertainty (e.g. confidence intervals)
☐	☒	For null hypothesis testing, the test statistic (e.g. *F*, *t*, *r*) with confidence intervals, effect sizes, degrees of freedom and *P* value noted<br>*Give P values as exact values whenever suitable.*
☐	☒	For Bayesian analysis, information on the choice of priors and Markov chain Monte Carlo settings
☒	☐	For hierarchical and complex designs, identification of the appropriate level for tests and full reporting of outcomes
☐	☒	Estimates of effect sizes (e.g. Cohen's *d*, Pearson's *r*), indicating how they were calculated

Our web collection on statistics for biologists contains articles on many of the points above.

Software and code

Policy information about availability of computer code

Data collection	All data included in this study was collected from previous observational studies. We did not use any software for data collection.
Data analysis	The AE models were implemented, trained, and tested using PyTorch71 v. 1.7.1. All source code is available at https://github.com/abrinkk/psg-age-estimation, which includes instructions for use.

For manuscripts utilizing custom algorithms or software that are central to the research but not yet described in published literature, software must be made available to editors and reviewers. We strongly encourage code deposition in a community repository (e.g. GitHub). See the Nature Research guidelines for submitting code & software for further information.

Data

Policy information about availability of data

All manuscripts must include a data availability statement. This statement should provide the following information, where applicable:

- Accession codes, unique identifiers, or web links for publicly available datasets
- A list of figures that have associated raw data
- A description of any restrictions on data availability

Polysomnography data included in this study was subject to data sharing agreement but is available upon reasonable request from E.M. (for STAGES and SSC), P.E.P (WSC), or upon request from the NSRR (SHHS, MrOS, CFS, and HomePAP).

Field-specific reporting

Please select the one below that is the best fit for your research. If you are not sure, read the appropriate sections before making your selection.

☒ Life sciences ☐ Behavioural & social sciences ☐ Ecological, evolutionary & environmental sciences

For a reference copy of the document with all sections, see nature.com/documents/nr-reporting-summary-flat.pdf

Life sciences study design

All studies must disclose on these points even when the disclosure is negative.

Sample size	We included all relevant data (n = 13,946) available to us either through clinics directly involved, academic colloborations, or the NSRR.
Data exclusions	Across the cohorts, PSGs were excluded if the subjects age was unknown, the recording was a CPAP split-night, the recording included less than 3 hours of sleep, or if more than two of the PSG signals were missing. All included PSG recordings are listed along with the code.
Replication	The age estimation models were optimized multiple times on the same training data before submission. Models were tested on unbiased and independent test sets.
Randomization	PSGs were sampled between the training, validation, and test set based on the described algorithm in Supplementary Table 16. In case of ties, which happens frequently, PSGs were sampled randomly.
Blinding	The allocation of samples was randomized and all data was recorded and scored before. The test was not used in any model development.

Reporting for specific materials, systems and methods

We require information from authors about some types of materials, experimental systems and methods used in many studies. Here, indicate whether each material, system or method listed is relevant to your study. If you are not sure if a list item applies to your research, read the appropriate section before selecting a response.

Materials & experimental systems

n/a	Involved in the study	
☒	☐	Antibodies
☒	☐	Eukaryotic cell lines
☒	☐	Palaeontology and archaeology
☒	☐	Animals and other organisms
☐	☒	Human research participants
☒	☐	Clinical data
☒	☐	Dual use research of concern

Methods

n/a	Involved in the study	
☒	☐	ChIP-seq
☒	☐	Flow cytometry
☒	☐	MRI-based neuroimaging

Human research participants

Policy information about studies involving human research participants

Population characteristics	To facilitate the development and testing of the AE models, the combined data was split into a training set (n = 2,500), a validation set (n = 200), and a test set (n = 11,699). Supplementary Fig. 3 shows the distribution of age and cohorts across the training, validation, and test set. Supplementary Table 17 shows the distribution of routinely used PSG metrics across the included cohorts.
Recruitment	Here we describe how participants were recruited from the included cohorts.<br><br>The Cleveland Family Study: Families were identified from a single member who had laboratory-confirmed sleep apnea. Control families were recruited using neighborhood families. For more information see: https://doi.org/10.1164/ajrccm.159.5.9809079<br><br>The MrOS Sleep Study: Subjects were enrolled from six different clinical sites in the US: Alabama, Minnesota, Oregon, Pennsylvania, and California. For more information see: https://doi.org/10.1016/j.cct.2005.05.005<br><br>The Wisconsin Sleep Cohort: Wisconsin state employees were recruited to study longitudinal patterns in sleep and health outcomes. For more information see: https://pophealth.wisc.edu/research/the-wisconsin-sleep-cohort/<br><br>The Stanford Technology Analytics and Genomics of Sleep: This is a a prospective cross-sectional, multi-site study involving 11 data collection sites from six centers in the US: Stanford University, Bogan Sleep Consultants, Geisinger, Mayo Clinic, MedSleep, and St. Luke's Hospital. For more information see: https://doi.org/10.1093/sleep/zsz067.321

The Stanford Sleep Cohort: Participants were patients who were recruited from the Stanford Sleep Clinic. For more information see: https://doi:10.1001/jamaneurol.2013.1589

The Sleep Heart Health Study: Participants were recruited from established cohorts directed at studying sleep and cardiovascular diseases in several ethnic groups of at least 40 years of age. The incident rate of cardiovascular disease is higher in this study. For more information see: https://doi.org/10.1093/sleep/20.12.1077

The Home Positive Airway Pressure Study: New referrals with high risk of obstructive sleep apnea were recruited from 7 sleep centers across the US: Case Western Reserve University affiliates (University Hospitals, MetroHealth Medical Center, and Cleveland Clinic) in Cleve-land, OH; Northwestern University in Chicago, IL; University of Wisconsin in Madison, WI; University of Minnesota in Minneapolis, MN; and University of Washington in Seattle, WA. For more information see: https://doi.org/10.5665/sleep.1870

Ethics oversight

Here we describe the ethics oversight for each included cohort.

The Cleveland Family Study: The institutional review committee at the University Hospitals Case Medical Center approved the study protocol, and all participants provided written informed consent.

The MrOS Sleep Study: All men provided written informed consent. The study was approved by the Institutional Review Board at each site.

The Wisconsin Sleep Cohort: Informed consent was obtained from all subjects. TheInstitutional Review Board at the University of Wisconsin-Madison Health Sciences approved this study.

The Stanford Technology Analytics and Genomics of Sleep: Informed consent was obtained from all participants. The study was approved by the Institutional Review Board at each site.

The Stanford Sleep Cohort: All participants gave written informed consent. The Stanford iInstitutional Review Board approved the study for patients included in the Stanford databases.

The Sleep Heart Health Study: The protocol of the study was approved by the Institutional Review Board of each participating institution and signed informed consent was provided by all subjects.

The Home Positive Airway Pressure Study: Informed consent was gathered from each participant and the project was approved by the Institutional Review Board at each site.

Note that full information on the approval of the study protocol must also be provided in the manuscript.
